# A cancer mutation promotes EphA4 oligomerization and signaling by altering the conformation of the SAM domain

**DOI:** 10.1016/j.jbc.2021.100876

**Published:** 2021-06-15

**Authors:** Taylor P. Light, Maricel Gomez-Soler, Zichen Wang, Kelly Karl, Elmer Zapata-Mercado, Marina P. Gehring, Bernhard C. Lechtenberg, Taras V. Pogorelov, Kalina Hristova, Elena B. Pasquale

**Affiliations:** 1 Department of Materials Science and Engineering, Institute for NanoBioTechnology, Johns Hopkins University, Baltimore, Maryland, USA; 2 Cancer Center, Sanford Burnham Prebys Medical Discovery Institute, La Jolla, California, USA; 3 Department of Chemistry, Center for Biophysics and Quantitative Biology, Beckman Institute for Advanced Science and Technology, and National Center for Supercomputing Applications, School of Chemical Sciences, University of Illinois at Urbana−Champaign, Urbana, Illinois, USA; 4 Program in Molecular Biophysics, Institute for NanoBioTechnology, Johns Hopkins University, Baltimore, Maryland, USA

**Keywords:** receptor tyrosine kinase, phosphorylation, melanoma, molecular dynamics, FRET, ALS, amyotrophic lateral sclerosis, DMEM, Dulbecco’s modified eagle medium, FIF, fluorescence intensity fluctuation, FRET, Föster resonance energy transfer, FSI, fully quantified spectral imaging, HRP, horseradish peroxidase, LDS, lithium dodecyl sulfate, MD, molecular dynamics, MSE, mean squared error, PBS, phosphate buffered saline, RMSD, root mean square deviation, SAM, sterile alpha motif, SASA, solvent accessible surface area

## Abstract

The Eph receptor tyrosine kinases and their ephrin ligands regulate many physiological and pathological processes. EphA4 plays important roles in nervous system development and adult homeostasis, while aberrant EphA4 signaling has been implicated in neurodegeneration. EphA4 may also affect cancer malignancy, but the regulation and effects of EphA4 signaling in cancer are poorly understood. A correlation between decreased patient survival and high EphA4 mRNA expression in melanoma tumors that also highly express ephrinA ligands suggests that enhanced EphA4 signaling may contribute to melanoma progression. A search for EphA4 gain-of-function mutations in melanoma uncovered a mutation of the highly conserved leucine 920 in the EphA4 sterile alpha motif (SAM) domain. We found that mutation of L920 to phenylalanine (L920F) potentiates EphA4 autophosphorylation and signaling, making it the first documented EphA4 cancer mutation that increases kinase activity. Quantitative Föster resonance energy transfer and fluorescence intensity fluctuation (FIF) analyses revealed that the L920F mutation induces a switch in EphA4 oligomer size, from a dimer to a trimer. We propose this switch in oligomer size as a novel mechanism underlying EphA4-linked tumorigenesis. Molecular dynamics simulations suggest that the L920F mutation alters EphA4 SAM domain conformation, leading to the formation of EphA4 trimers that assemble through two aberrant SAM domain interfaces. Accordingly, EphA4 wild-type and the L920F mutant are affected differently by the SAM domain and are differentially regulated by ephrin ligand stimulation. The increased EphA4 activation induced by the L920F mutation, through the novel mechanism we uncovered, supports a functional role for EphA4 in promoting pathogenesis.

The EphA4 receptor is a member of the Eph family of receptor tyrosine kinases, which is known to control a variety of cellular functions such as cell adhesion, migration, and invasion by modifying the organization of the actin cytoskeleton (1, 2, 3). EphA4 is primarily expressed in the nervous system, where it plays a critical role in neural development and regulates synaptic plasticity in the adult brain (3, 4, 5). Furthermore, EphA4 has been implicated in neurodegenerative diseases such as amyotrophic lateral sclerosis (ALS) and Alzheimer’s disease (6, 7). EphA4 is also expressed in nonneural tissues, where its activities are less well understood. In addition, EphA4 has been proposed to play a role in different cancers, including melanoma (8, 9), breast cancer (10, 11, 12, 13), glioma (14), hematologic malignancies (15, 16), pancreatic cancer (17, 18), prostate cancer (19, 20), and lung cancer (21) as well as in resistance to chemotherapy and radiotherapy (15, 22, 23). Furthermore, EphA4 somatic mutations have been identified in a number of tumor types (cbioportal.org). Interestingly, in melanoma, a cancer derived from cells of neural crest origin, EphA4 is preferentially mutated in tumors lacking major driver mutations (8). This suggests the potential clinical relevance of EphA4 mutations as part of a constellation of gene mutations cooperating to promote melanoma progression in tumors lacking a dominant driver mutation. However, the precise role of EphA4 in cancer progression and the mechanisms underlying the potential oncogenic activity of EphA4 are poorly understood.

The domain architecture of EphA4 is similar to that of most other receptor tyrosine kinases, with an extracellular region, a single-pass transmembrane helix, and an intracellular region (24). The extracellular region contains the ligand-binding domain at the N-terminus, a Sushi domain, an epidermal growth factor-like domain, and two fibronectin type III domains (Fig. 1*C*) (25). The EphA4 intracellular region comprises a juxtamembrane segment, the catalytic tyrosine kinase domain, and a sterile alpha motif (SAM) domain followed by a PDZ domain binding motif at the C-terminus (26). EphA4 is known to dimerize upon ephrin ligand binding (27). In the dimers, EphA4 molecules cross-phosphorylate each other, mainly on selected tyrosines in the juxtamembrane segment and in the activation loop of the kinase domain (phosphosite.org) (28).

**Figure 1:**
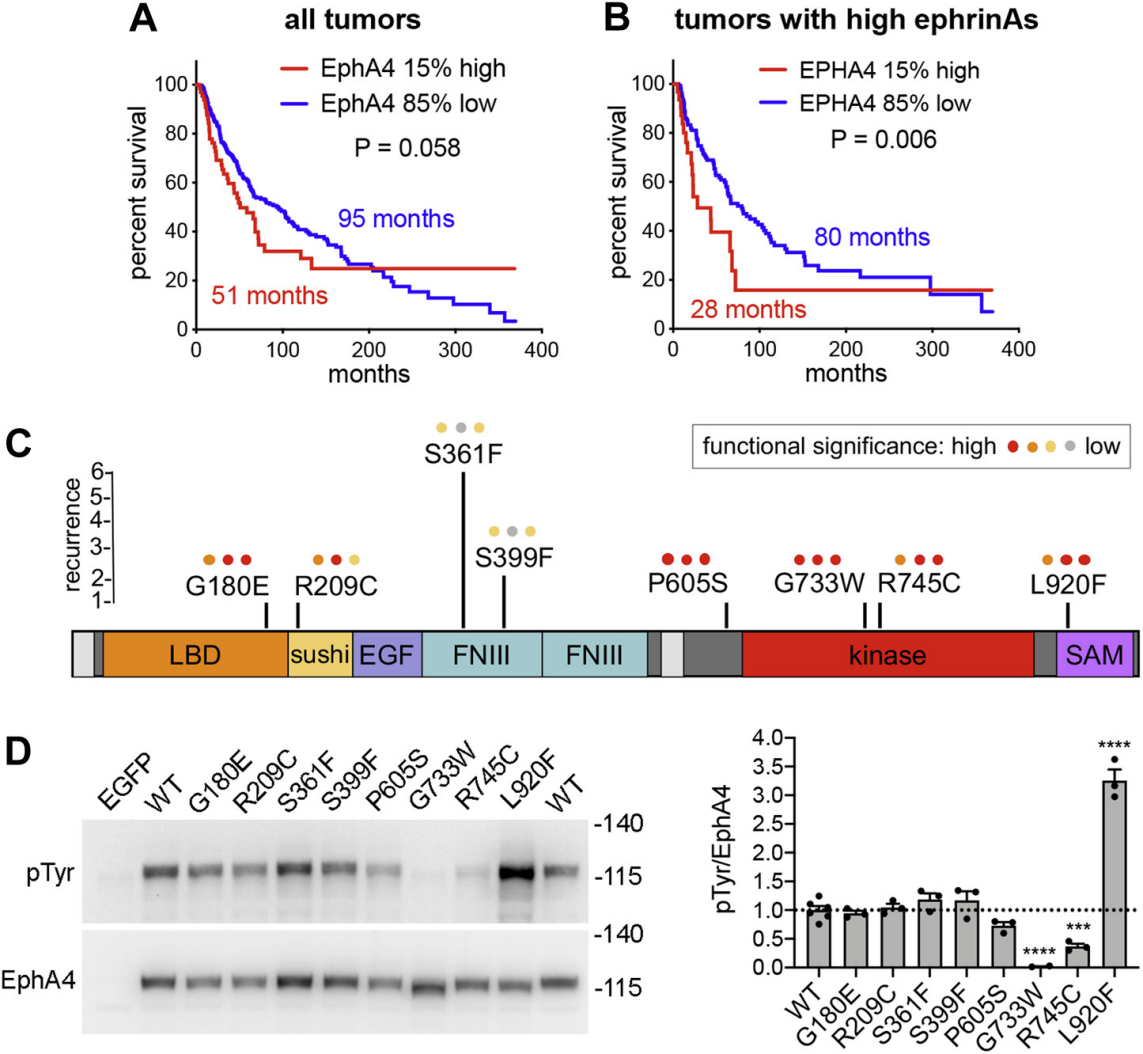
**Correlation of EphA4 expression with patient survival and EphA4 mutations in melanoma.***A*, the correlation between high (top 15%) EphA4 mRNA expression and decreased overall patient survival does not reach statistical significance when considering all melanoma tumors. *B*, high EphA4 expression in the subset of tumors with highest (top 15%) mRNA expression of one or more of the five ephrinA ligands significantly correlates with decreased patient survival. In both *A* and *B*, the 15% of tumor samples with highest EphA4 expression were compared to the 85% remaining tumor samples. *B* includes for each ephrinA ligand the 15% of tumors with highest expression, for a total of ∼45% of all the tumors. mRNA expression z-scores relative to all samples (log RNA Seq V2 RSEM) from the TCGA Firehose Legacy skin cutaneous melanoma dataset (n = 472 tumor samples with mRNA expression data) were used for analysis. Median survival times are indicated in the graphs and *p* values were calculated using the log-rank Mantel–Cox test. *C*, the location of the eight EphA4 melanoma mutations analyzed is shown in relation to the EphA4 domain structure. The mutations were selected for further investigation from 12 skin melanoma studies available in the cBioPortal website (cbioportal.org). The height of the *black vertical lines* indicates the number of tumors with that particular mutation. The *colored dots* above the name of each mutation indicate the prediction of functional significance according to three prediction programs: Mutation Assessor, SIFT and PolyPhen-2 (cbioportal.org). The EphA4 signal peptide and transmembrane helix are shown in *light gray* and linkers are shown in *dark gray*, including the juxtamembrane segment containing the P605S mutation. *D*, EphA4 WT, the eight EphA4 mutants, and EGFP as a control were transiently expressed in HEK293 cells and cell lysates were probed by immunoblotting with antibodies to phosphotyrosine (pTyr) and to EphA4. The bar graph shows averages and standard errors from quantifications of three experiments (individual values from each experiment are shown as *dots*). ∗∗∗*p* < 0.001 and ∗∗∗∗*p* < 0.0001 for the comparison with WT by one-way ANOVA. EGF, epidermal growth factor-like domain; FNIII, fibronectin type III domain; LBD, ligand-binding domain; kinase, kinase domain; SAM, sterile alpha motif domain; sushi, sushi domain.

We show here that the L920F gain-of-function mutation identified in a melanoma metastasis (cbioportal.org) involves a leucine in the EphA4 SAM domain that is conserved in all Eph receptors (29). The SAM domain can contribute to Eph receptor dimerization/oligomerization as well as mediate interactions with cytosolic adaptor proteins that transduce downstream signaling responses (30, 31, 32). Therefore, to understand the effect of the L920F mutation on EphA4 function, we characterized and compared the activation and oligomerization of WT and L920F mutant EphA4. These studies yield mechanistic insights into the importance of the SAM domain in EphA4 activation and how the L920F mutation can lead to dysregulated EphA4 signaling and disease.

## Results

### EphA4 expression and mutations in melanoma

Analysis of the TCGA collection of skin cutaneous melanoma samples revealed that high EphA4 mRNA expression correlates with decreased patient survival in tumors that also exhibit high expression of one or more of the five ephrinA ligands, which bind EphA4 with high affinity (33) (Fig. 1, *A* and *B*). In particular, ephrinA3 and ephrinA4 are the ephrinA ligands most frequently highly expressed in the tumors with high EphA4 mRNA analyzed in Figure 1*B* (in 69% and 62% of the tumors, respectively, with 54% of the tumors expressing both ephrins). This suggests that high EphA4 activation may promote melanoma malignancy and, therefore, that EphA4 activating mutations may play a role in melanoma progression.

EphA4 is mutated in approximately 4% of the 1499 skin melanoma patients for which data are available in cBioPortal (cbioportal.org). These mutations include 47 missense mutations (of which seven are recurrent), four nonsense mutations (two recurrent), and two splice site mutations. We analyzed eight of the EphA4 missense mutations, which were chosen based on their predicted functional impact or recurrence in more than one sample and location in different regions of the receptor (Fig. 1*C*). To determine the effects of the mutations on EphA4 activation, we engineered the different EphA4 mutant constructs with an N-terminal FLAG tag, expressed them by transient transfection in HEK293 cells, and determined their level of autophosphorylation on tyrosine residues. Immunoblot analysis of cell lysates showed that EphA4 WT is substantially tyrosine phosphorylated (Fig. 1*D*), as expected because the high receptor expression in transiently transfected cells presumably causes EphA4 dimerization and autophosphorylation (34, 35). Most of the mutations did not significantly affect EphA4 tyrosine phosphorylation. The G733W and R745C mutations in the kinase domain abolished or greatly decreased, respectively, EphA4 tyrosine phosphorylation. Consistent with this effect, the R745 mutation is part of the HRDLAA motif in subdomain VI, which is highly conserved in tyrosine kinase domains and known to be important for catalytic activity. In contrast, the L920F mutation affecting a conserved leucine residue in the EphA4 SAM domain stood out for its ability to drastically increase EphA4 tyrosine phosphorylation (Fig. 1*D*), as expected for a mutation that promotes receptor activation. We therefore focused on the further characterization of the EphA4 L920F mutant.

### The L920F mutation promotes EphA4 activation and signaling

**Figure 2:**
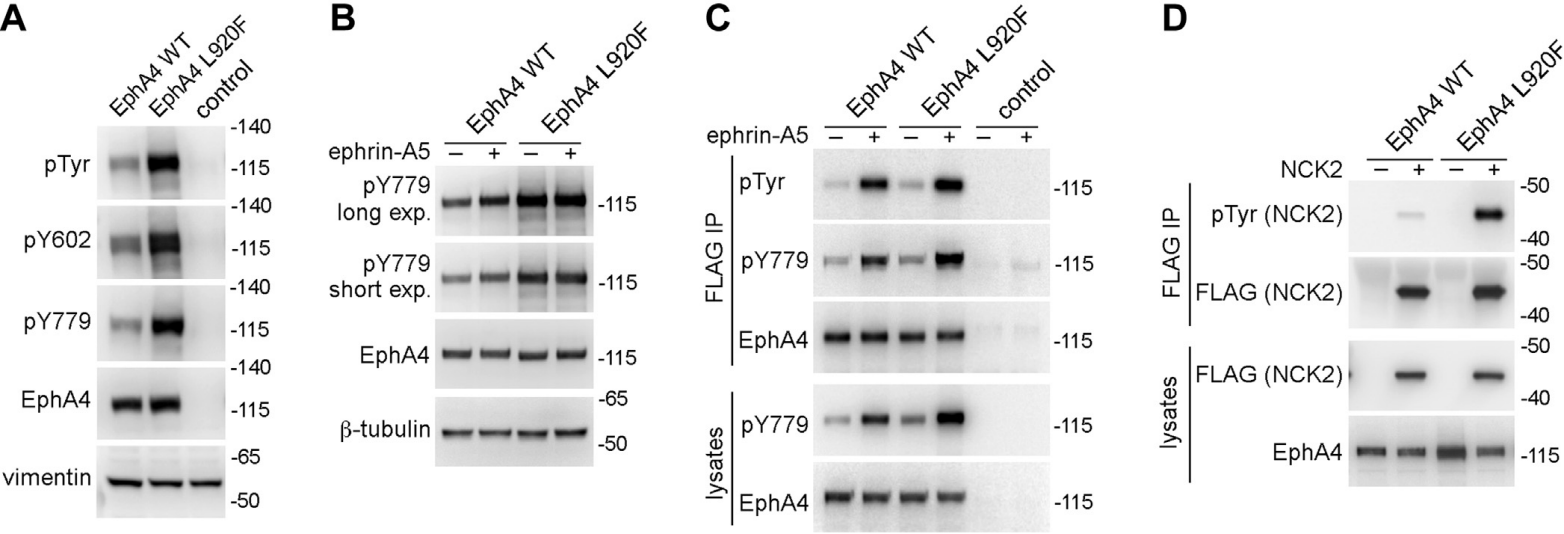
**The L920F mutation in the EphA4 SAM domain promotes receptor tyrosine phosphorylation and activation.***A*, HEK293 cells were transiently transfected with constructs encoding EphA4 WT, the EphA4 L920F mutant, or EGFP as a control. Cell lysates were probed by immunoblotting with antibodies to phosphotyrosine (pTyr), the Y602 and Y779 EphA4 phosphorylation sites, EphA4, and vimentin as a loading control. *B*, HEK293 cells were transiently transfected as indicated and treated for 10 min with 0.5 μg/ml ephrinA5-Fc (+) or Fc as a control (−). Cell lysates were probed by immunoblotting with antibodies to the Y779 EphA4 phosphorylation site (short and long exposures are shown), EphA4, and β-tubulin as a loading control. *C*, HEK293 cells were stably transfected with constructs encoding FLAG-tagged EphA4 WT or L920F mutant, or EGFP as a control, and treated for 10 min with 0.5 μg/ml ephrinA5-Fc (+) or Fc as a control (−). FLAG immmunoprecipitates and cell lysates were probed by immunoblotting with the indicated antibodies. *D*, HEK293 cells were transiently transfected with constructs encoding Strep-tagged EphA4 WT or L920F mutant with or without FLAG-tagged NCK2. FLAG immunoprecipitates and cell lysates were probed by immunoblotting with the indicated antibodies.

The effect of the L920F mutation on EphA4 activation was confirmed by using phospho-specific antibodies recognizing two conserved major autophosphorylation sites, Y602 in the juxtamembrane segment and Y779 in the activation loop (Fig. 2*A*). The phosphorylation of these two key tyrosine residues is involved in EphA4 activation and signaling (28, 36). Therefore, the increased phosphorylation of the EphA4 L920F mutant on these two residues supports the notion that the L920F mutation increases ephrin ligand-independent EphA4 activation under conditions of overexpression.

Treatment of transiently transfected HEK293 cells with the EphA4 ligand ephrinA5-Fc increased EphA4 WT tyrosine phosphorylation, but not the already very high tyrosine phosphorylation of the EphA4 L920F mutant (Fig. 2*B*). We also examined stably transfected cells, in which receptor expression is lower and therefore the constitutive tyrosine phosphorylation of the EphA4 L920F mutant is not as pronounced (Fig. 2*C*). Treatment with ephrinA5-Fc markedly increased tyrosine phosphorylation of both EphA4 WT and L920F (Fig. 2*C*), indicating that EphA4 L920F can still respond to ligand stimulation.

To determine whether the increased tyrosine phosphorylation of the EphA4 L920F mutant corresponds to increased signaling activity, we examined the SH2 domain-containing adaptor protein NCK2, which is a known EphA4 substrate and downstream effector (37, 38, 39, 40). Coexpression of FLAG-tagged NCK2 with the EphA4 L920F mutant in transiently transfected HEK293 cells caused much higher NCK2 tyrosine phosphorylation than coexpression with EphA4 WT (Fig. 2*D*). This is consistent with a higher kinase activity of the EphA4 L920F mutant compared with WT, which presumably leads to both increased interaction of NCK2 with EphA4 L920F (due to higher phosphorylation of the two conserved juxtamembrane motifs reported to bind the NCK2 SH2 domain) and increased phosphorylation of NCK2 tyrosines by the activated EphA4 L920F mutant (37, 38, 39, 40).

### The L920F mutation perturbs the conformation of the EphA4 SAM domain

**Figure 3:**
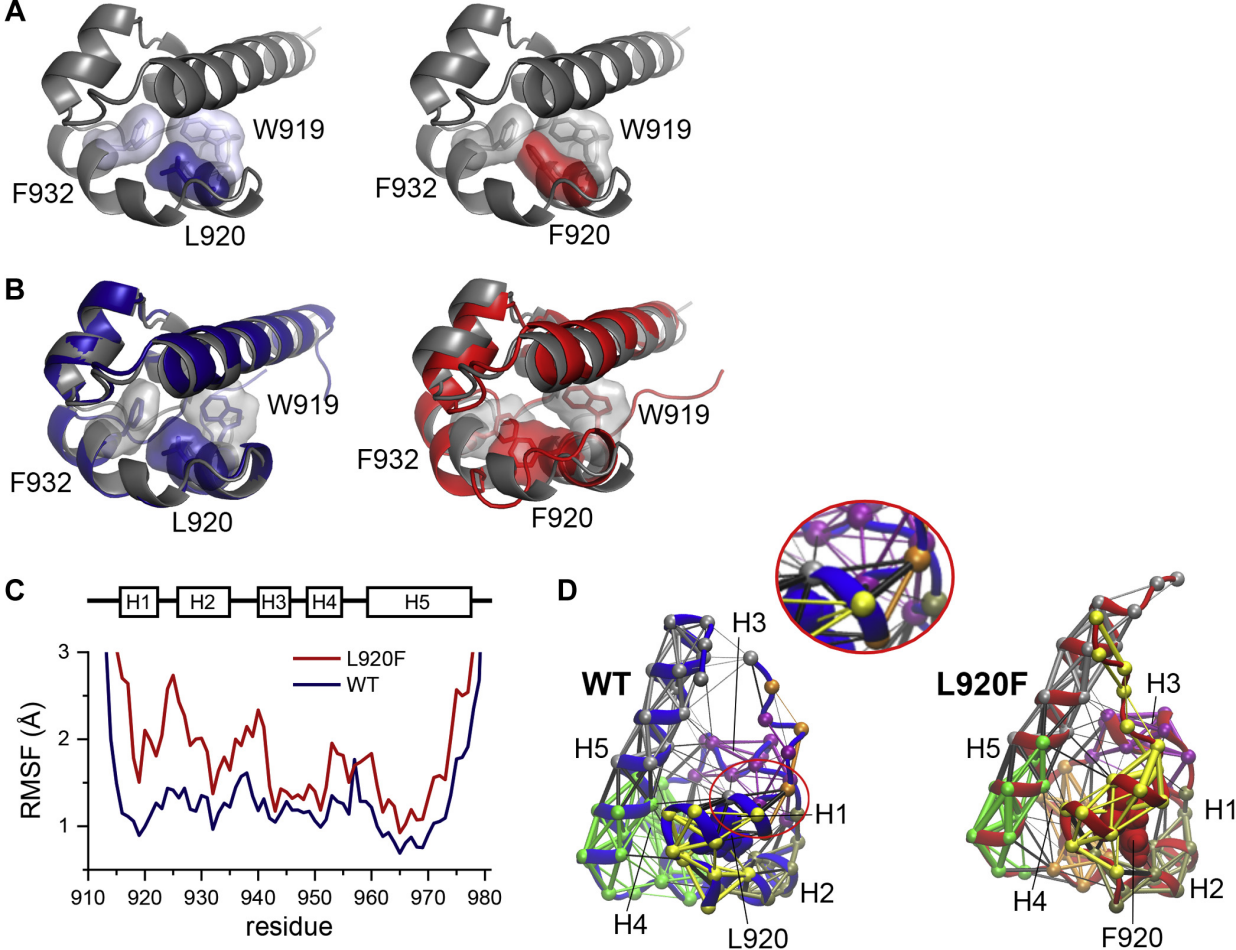
**All-atom MD simulations suggest that the EphA4 L920F mutation introduces local and global structural perturbations.***A*, structure of the EphA4 WT SAM domain. (*left*; PDB ID: 1B0X; (30)) and model of the L920F mutant (*right*), obtained by direct substitution of L920 with phenylalanine, in *ribbon* representation with the indicated residues shown as *sticks* and as a molecular surface. The model illustrates how W919 and F932 clash with a phenylalanine at position 920. *B*, representative structures obtained from MD simulations of the EphA4 WT SAM domain (*left*) and EphA4 L920F SAM domain (*right*) are aligned with the EphA4 SAM domain crystal structure (*gray*) by minimizing backbone RMSD values. *C*, the RMSF values calculated using the crystal structure coordinates as the reference are plotted for each residue in the EphA4 WT and L920F SAM domains over the course of the MD simulations, each averaged over three replicates. A schematic of the SAM domain helix positions is shown above the figure. *D*, dynamic network analysis for the EphA4 WT and L920F mutant SAM domain structures. Nodes, indicated by *spheres*, highlight the α-carbon atoms and the thickness of the edges (lines connecting the nodes) is proportional to the correlation of atomic motion in space and time. The WT L920 or mutant F920 is rendered with atoms as spheres. Six communities (sets of residues that exhibit coordinated motion) are shown in different colors: community 1 in *yellow*, community 2 in *olive*, community 3 in *purple*, community 4 in *orange*, community 5 in *green*, and community 6 in *gray*. Edges drawn within the same community are colored according to that community and edges drawn between nodes of different communities are colored *black*. The α-helices (H1 through H5) are indicated and the portion of H1 circled in *red* in the WT SAM domain is also shown as an enlargement to highlight the different communities in this α-helix.

The solved structure of the EphA4 SAM domain (PDB ID: 1B0X) (30) shows that the side chain of L920 in helix 1 is buried in the interior of the domain, in close proximity to W919 in helix 1 and F932 in helix 2 (Fig. 3*A*, left). The larger phenylalanine cannot fit in the available space, as shown by modeling a phenylalanine in the crystal structure, which shows that the molecular surface of F920 clashes with F932 (Fig. 3*A*, right). Therefore, the L920F mutation is expected to alter the conformation of the EphA4 SAM domain to alleviate the steric strain, thus potentially affecting receptor functional properties.

To understand the effects of the L920F mutation on the structure and dynamics of the EphA4 SAM domain, we performed all-atom molecular dynamics (MD) simulations of the EphA4 WT and L920F SAM domains, using the crystal structure of the SAM domain monomer as a starting point for the simulations. The conformation of the EphA4 WT SAM domain after the MD simulations was largely unperturbed compared with the crystal structure (Fig. 3*B*, left), as shown by the minimal variations in root mean square deviations (RMSDs) (∼1 Å; Fig. S1). The stable structural ensemble obtained from the MD simulations with the WT SAM domain also demonstrates use of the appropriate force field.

We observed larger structural perturbations in the L920F mutant SAM domain, where the relative positions of the helices were slightly shifted (Fig. 3*B*, right). Indeed, root-mean-square fluctuations (RMSFs) for each residue in the MD trajectory with respect to the crystal structure as reference revealed not only nearly twice as large RMSF values for the region around the mutated L920 compared with WT but also higher global fluctuations for the L920F mutant than for WT (Fig. 3*C*). The largest differences between the L920F MD structure and the WT MD and crystal structures were observed in helices 1 to 3 (H1–3), spanning residues 915 to 940 (Fig. 3*C*). Thus, as expected, the residues in the core of the EphA4 SAM domain rearrange to accommodate the size of the phenylalanine residue. The structural perturbations due to the mutation expose the EphA4 SAM domain core to the solvent, as determined by measuring solvent accessible surface area (SASA) for core residues L920/F920 and F932 over the entire MD simulation trajectory. In contrast, surface residues W919 and H945 used as controls remained similarly accessible to the solvent (Fig. S2). The structural instability induced by the L920F mutation is evident not only from the large variation in the SASA values for this residue but also from the haphazard RMSD changes in the entire SAM domain over the course of the simulations (Fig. S1).

To study the conformational coupling within the folded SAM domains, we performed dynamic network analysis and community clustering (Fig. 3*D*). Edges (represented by lines) were drawn between nonadjacent nodes (represented by spheres located on the α-carbon atoms of each residue in the structure). The thickness of the lines is proportional to the degree of linear correlation in the positions of connected residue pairs. Residues were classified into communities so that residues within the same community show motions that are more correlated with each other than with residues in other communities. Six communities were observed for both the WT and L920F SAM domain structures. Most of the classified communities are similar in the two structures, with the notable exception of helix 1 containing the mutated residue 920. In the WT structure, parts of helix 1 were classified as extensions of nearby communities, as illustrated by the multiple colors of helix 1 (Fig. 3*D*, left and enlargement). This suggests that interhelical interactions could potentially stabilize the SAM domain fold. In contrast, the L920F mutation diminishes coupling between helix 1 and nearby helices, as demonstrated by the relatively fewer connections between communities compared with the WT SAM domain (Fig. 3*D*, right). Furthermore, the entire helix 1 was classified as one independent community (yellow) in the L920F SAM domain. It can be inferred from this heavily interconnected helix 1 community that the L920F mutation reshapes helix 1 into a stable autonomous structure with locally confined motions. We also observed differences in other helices. The community extending from helix 4 into a portion of helix 5 in the WT structure (green) becomes decoupled in the L920F structure, where helix 4 becomes an independent community (orange) separate from helix 5 (Fig. 3*D*). Furthermore, helices 2, 3, and 5 are stabilized by the L920F mutation, as depicted by the thicker edges within the communities compared with those in the WT SAM domain. Thus, while interhelical coupling is diminished slightly by the L920F mutation, the conformational change induced by the mutation stabilizes the individual helices, which in turn may stabilize the SAM domain tertiary structure.

### The EphA4 L920F mutation promotes EphA4 oligomerization

**Figure 4:**
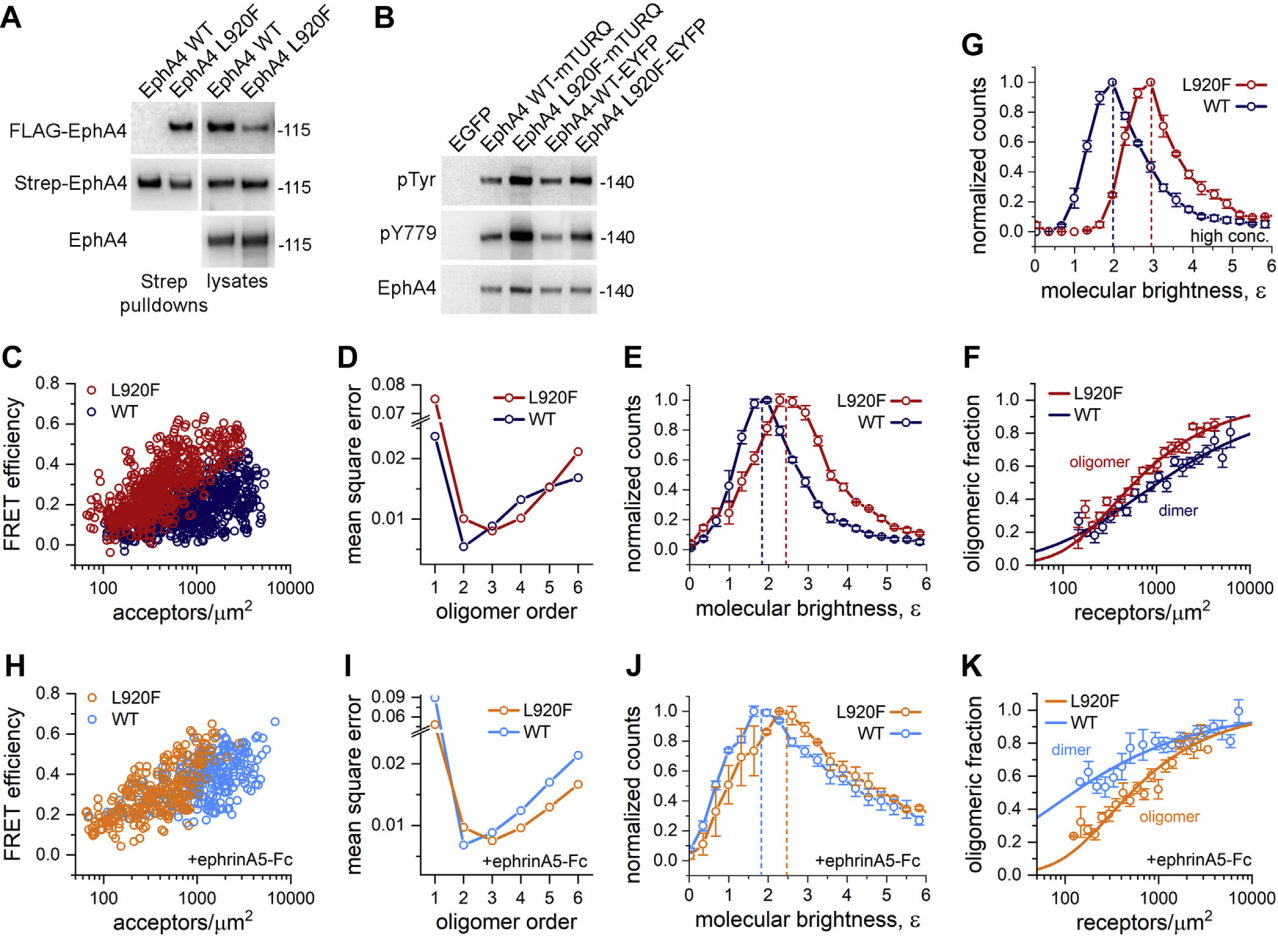
**The L920F mutation induces EphA4 oligomerization.***A*, HEK293 cells were transiently cotransfected with FLAG- or Strep-tagged EphA4 WT or L920F mutant. Cell lysates were subjected to pull-down with Strep-Tactin beads and the proteins bound to the beads were eluted, split into two aliquots and each aliquot was probed by immunoblotting with antibodies to the FLAG tag to detect FLAG-EphA4 or to the Strep tag to detect Strep-EphA4. Different aliquots of the lysates were also probed as indicated. *B*, HEK293 cells were transiently cotransfected with EphA4 (WT or L920F mutant) fused to C-terminal mTURQ or EYFP fluorescent proteins. Cell lysates were probed by immunoblotting with antibodies to phosphotyrosine (pTyr), the Y779 EphA4 phosphorylation site, and EphA4. *C*, FRET efficiencies measured for EphA4-mTURQ and EphA4-EYFP, WT, and L920F mutant, in the absence of ligand using the FSI-FRET method. *D*, mean square error (MSE) values for best-fit oligomerization models ranging from monomers (*n* = 1) to hexamers (*n* = 6). The minimum MSE indicates the model that best describes the data. *E*, FIF measurements performed in HEK293 cells expressing EphA4 WT or L920F. Shown are histograms of the measured molecular brightness (ε), which scales with the oligomer size. The brightness values corresponding to the histogram maxima are indicated by the *dotted lines*. *F*, dimeric or oligomeric fractions calculated from the FSI-FRET data are plotted as a function of total receptor concentration for EphA4 WT and L920F. The symbols represent the binned oligomeric fractions and their standard errors. The *solid lines* represent the best fit curves for monomer–dimer or monomer–oligomer equilibrium. Although lower maximal EphA4 L920F acceptor expression was achieved than for WT (panel *C*), a complete oligomerization curve was obtained. The dissociation constant (or apparent dissociation constant in the oligomer case) is determined as the receptor concentration at which the oligomeric fraction is 0.5 (50%; see Table 1). *G*, high-concentration FIF histograms for EphA4 WT and L920F mutant, generated by using the data from *E* only for receptor concentrations higher than 3000 receptors/μm^2^. By removing low-concentration data, the monomer populations are not significantly present and thus the maximum of the histogram more accurately indicates receptor oligomer size, ε = 2 (dimer) for EphA4 WT and ε = 3 (trimer) for the L920F mutant. *H*, FRET efficiencies as a function of acceptor concentration measured for EphA4 WT and L920F in the presence of the ephrinA5-Fc ligand. *I*, MSE values for EphA4 WT and EphA4 L920F with ephrinA5-Fc. *J*, FIF histograms for EphA4 WT and L920F mutant in the presence of ephrinA5-Fc. The maxima of the histograms are indicated by *dotted lines*. *K*, oligomerization curves for EphA4 WT and the EphA4 L920F mutant in the presence of ephrinA5-Fc (see Table 1). Although lower maximal EphA4 L920F acceptor expression was achieved than for WT (panel *H*), a complete oligomerization curve was obtained. FRET, Föster resonance energy transfer.

The SAM domain can affect Eph receptor oligomerization (including dimerization and/or the formation of larger oligomers) (30, 35, 41, 42, 43). Due to its location in the SAM domain, the L920F mutation could therefore affect the association of EphA4 molecules with each other. To address this possibility, we first performed pull-down experiments using lysates from HEK293 cells transiently cotransfected with constructs encoding EphA4 with N-terminal Strep or FLAG fusion tags. Immunoblot analyses did not reveal detectable FLAG-EphA4 WT associated with the pulled down Strep-EphA4 WT (Fig. 4*A*). In contrast, the FLAG-EphA4 L920F mutant was readily detectable in pull-downs of the Strep-EphA4 L920F mutant.

While the Eph receptors are known to form larger oligomers when they bind ephrin ligands, both EphA2 and EphA3 can also form dimers in the absence of ligand (34, 35). Thus, we used a Föster resonance energy transfer (FRET) approach to determine whether EphA4 can also dimerize in the absence of ligand and to examine the effects of the L920F mutation on EphA4 oligomerization. For these FRET experiments, EphA4 was tagged at its C-terminus with one of two fluorescent proteins that constitute a FRET pair, mTurquoise (mTURQ, the donor) or enhanced yellow fluorescent protein (EYFP, the acceptor), attached through a flexible (GGS)_5_ linker. After verifying that the L920F mutation also increases the autophosphorylation of fluorescently tagged EphA4 (Fig. 4*B*), we transiently cotransfected EphA4-mTURQ and EphA4-EYFP in HEK293 cells for fully quantified spectral imaging FRET (FSI-FRET) analysis.

Cells were imaged using a spectrally resolved two-photon microscope to acquire complete FRET and acceptor spectra. These spectra were analyzed with FSI-FRET software to calculate the two-dimensional concentrations of donor-labeled and acceptor-labeled EphA4 in micron-sized areas of the plasma membrane as well as the FRET efficiencies, which were corrected for nonspecific proximity FRET contributions (44, 45). The FRET efficiencies measured for EphA4 WT and the L920F mutant in the absence of ligand increase as a function of acceptor concentration (Fig. 4*C*), which indicates that EphA4 self-association increases with the receptor concentration in the plasma membrane, in accordance with the law of mass action. Therefore, EphA4 can self-associate in the absence of ligand, as we have previously shown for EphA2 and EphA3 (34, 35, 42). We observed higher FRET efficiencies for the EphA4 L920F mutant than for EphA4 WT (Fig. 4*C*), suggesting increased receptor association due to the L920F mutation.

We interpreted the FSI-FRET data using thermodynamic models describing various oligomerization states (monomer, dimer, or higher-order oligomers), as previously described in detail (44, 45). This analysis allowed us to determine what type of oligomeric model (with oligomer order *n*) best fits the data by calculating and comparing the mean squared errors (MSEs) for the different oligomeric models. The minimum MSE value for EphA4 WT is at *n* = 2 (Fig. 4*D*), suggesting that EphA4 WT associates into dimers at the plasma membrane. On the other hand, the minimum MSE for the EphA4 L920F mutant is at *n* = 3, indicating the formation of oligomers larger than dimers (Fig. 4*D*).

To confirm that the L920F mutation induces the formation of higher order oligomers, we used fluorescence intensity fluctuation (FIF) analysis, a technique based on the analysis of molecular brightness in small regions of the plasma membrane (46). Molecular brightness, defined as the ratio between the variance of the fluorescence intensity and the mean fluorescence intensity within a small membrane region, is known to scale with oligomer size (46). We found that the distribution of molecular brightness values is shifted to higher values for the L920F mutant compared with EphA4 WT (Fig. 4*E*), confirming that the EphA4 L920F mutant forms larger oligomers. Since the FIF histograms shown in Figure 4*E* include data measured over a wide range of receptor concentrations, a mixed population of monomers and oligomers is included in the analysis. Thus, the peak in the brightness distribution for EphA4 WT is at molecular brightness <2, a value that is higher than expected for a monomer and lower than expected for a dimer. For EphA4 L920F, the peak is at molecular brightness >2, a value larger than expected for a dimer.

The fraction of oligomerized EphA4 molecules, calculated from the FRET data, can be plotted as a function of total EphA4 concentration in the plasma membrane in order to fit an oligomerization curve (Fig. 4*F*). For EphA4 WT, Equation 2 (see Experimental procedures) was used to obtain a best fit dimerization curve (Fig. 4*F*) and calculate the dissociation constant, *K*_*diss*_ (1050 ± 170 receptors/μm^2^; Table 1). For EphA4 L920F oligomers, Equation 5 was used to determine the best fit oligomerization curve, and we determined an apparent dissociation constant of ∼580 receptors/μm^2^ as the EphA4 concentration at which 50% of the molecules form oligomers (Fig. 4*F* and Table 1). Thus, the oligomeric fraction for EphA4 L920F is 50% at a lower receptor concentration than for EphA4 WT, indicating that the L920F mutation increases the propensity of EphA4 molecules to associate with each other.

**Table 1:** Summary of FSI-FRET experiments

Eph receptor	Ligand	K_*diss*_ (receptors/μm^2^)	ΔG (kcal/mol)
EphA4 WT	no ligand	1050 ± 170	−4.1 ± 0.1
EphA4 WT	ephrinA5-Fc	120 ± 50	−5.3 ± 0.2
EphA4 L920F	no ligand	∼580^a^	nd
EphA4 L920F	ephrinA5-Fc	∼510^a^	nd
EphA4 L920F-H945E	no ligand	∼1350^a^	nd
EphA4 L920F-H945E	ephrinA5-Fc	∼530^a^	nd
EphA4 H945E	no ligand	540 ± 80	−4.5 ± 0.1
EphA4 ΔSAM	no ligand	1120 ± 220	−4.0 ± 0.1
EphA2 WT^b^	no ligand	300 ± 70	−4.8 ± 0.1
EphA2 L913F	no ligand	90 ± 20	−5.5 ± 0.1

K_*diss*_ is the dissociation constant determined for the forms of EphA4 and EphA2 that form dimers. ΔG is the dimerization free energy calculated from the dissociation constant. Shown are the best fit values along with the 68% confidence intervals. FRET, Föster resonance energy transfer; nd, not determined.

a Apparent dissociation constants for EphA4 L920F and L920F-H945E, estimated from the best-fit oligomeric fraction curves. They correspond to the receptor concentrations at which 50% of the receptors are associated into oligomers.

b The values for K_*diss*_ and ΔG for EphA2 WT have been previously published (47).

To obtain FIF histograms without a major contribution from receptor monomers, we analyzed only the FIF data measured at high receptor concentrations (above 3000 receptors/μm^2^; Fig. 4*G*). At these concentrations, EphA4 is predominately oligomeric (with an oligomeric fraction of >65% for WT and >80% for the L920F mutant; Fig. 4*F*). The molecular brightness distributions for high receptor concentrations have their maxima at values of ε = 2 for EphA4 WT (indicating dimers) and ε = 3 for EphA4 L920F (indicating trimers; Fig. 4*G*). This further confirms that the L920F mutation alters EphA4 oligomer size from predominately dimeric to predominately trimeric. The higher oligomer order observed in both FRET and FIF experiments for EphA4 L920F compared with WT may explain the observed higher EphA4 L920F tyrosine phosphorylation and signaling observed in immunoblotting experiments (Figs. 1*D* and 2, *A* and *D*).

To further characterize the role of the SAM domain in EphA4 oligomerization, we analyzed an EphA4 mutant lacking this domain (EphA4 ΔSAM). This revealed that deletion of the SAM domain in EphA4 WT has a negligible effect on FRET efficiency (Fig. S3*A*) and that, similar to EphA4 WT, EphA4 ΔSAM associates into dimers in the plasma membrane (Fig. S3*B*). Furthermore, the dissociation constant for EphA4 ΔSAM dimers is 1120 ± 220 receptors/μm^2^, which is comparable with that for EphA4 WT dimers (Fig. S3*C* and Table 1). Thus, the SAM domain does not play a major role in the assembly of EphA4 oligomers on the cell surface, which is different from the roles reported for the SAM domains of the related EphA2 and EphA3 receptors (35, 42, 43). In contrast, comparison of FRET data for EphA4 ΔSAM and EphA4 L920F confirms the critical role of the L920F mutant SAM domain in both promoting EphA4 assembly in the absence of ligand and altering EphA4 oligomer size (Fig. S3, *D* and *E*).

We also examined the effect of ephrinA5-Fc on EphA4 oligomerization. Treatment of the transiently transfected HEK293 cells with this ligand increased FRET efficiencies for EphA4 WT, but not for the L920F mutant (Fig. 4, *C* and *H* and Fig. S4, *A* and *D*). The minimum MSE value in the presence of ephrinA5-Fc is at *n* = 2 for EphA4 WT and at *n* = 3 for EphA4 L920F (Fig. 4*I*), which suggests that EphA4 WT forms predominately dimers when ligand-bound, while the EphA4 L920F mutant forms predominately trimers. FIF experiments show that the maximum of the brightness distribution for EphA4 WT in the presence of ephrinA5-Fc occurs at molecular brightness <2, while the maximum for EphA4 L920F is at brightness >2 (Fig. 4*J*).

Comparison of the FIF brightness distributions for EphA4 L920F in the absence of ligand and EphA4 WT in the presence of ligand provides insight into the differential effects of the mutation and ligand binding on EphA4 oligomer size. The L920F mutation induces a switch in the EphA4 oligomer size from predominantly dimeric to predominantly trimeric (Fig. 4, *D* and *G*). In contrast, ligand binding to EphA4 WT preserves the dimer as the main oligomeric state (Fig. 4, *I* and *J* and Fig. S4*I*), but also promotes the formation of larger oligomers (Fig. S4*C*). Ligand binding to EphA4 L920F preserves the trimer as the main oligomeric state (Fig. 4, *I* and *J* and Fig. S4*I*) while also promoting the formation of larger oligomers (Fig. S4*F*). Therefore, the L920F mutant and ligand binding differentially affect EphA4 self-association.

Comparison of the oligomerization curves in the presence and in the absence of ephrinA5-Fc suggests that the ligand stabilizes EphA4 WT dimers but does not substantially affect the stability of EphA4 L920F trimers (Fig. S4, *B* and *E*). Consistent with this, in the presence of ephrinA5-Fc, the oligomeric fraction at low receptor concentrations is much higher for EphA4 WT than for the L920F mutant (Fig. 4*K*). Indeed, the dissociation constant for EphA4 WT in the presence of ephrinA5-Fc (120 ± 50 receptors/μm^2^) is approximately an order of magnitude lower than in the absence of ligand (Fig. S4*B* and Table 1). On the other hand, the apparent dissociation constant for the EphA4 L920F oligomerization curve in the presence of ligand (∼510 receptors/μm^2^) is similar to the value in the absence of ligand (Fig. S4*E* and Table 1). It should be noted that the oligomerization curves derived from FRET data do not provide information on the ability of ephrinA5-Fc to promote higher-order oligomers because they are based on a two-state model of oligomerization that assumes either a monomer–dimer equilibrium (for EphA4 WT) or a monomer–trimer equilibrium (for EphA4 L920F).

Taken together, the FRET and FIF data suggest that the increase in EphA4 WT tyrosine phosphorylation induced by ephrinA5-Fc (Fig. 2, *B* and *C*) is due to an increase in dimers and the formation of larger oligomers, while the increase in EphA4 L920F tyrosine phosphorylation mainly depends on the formation of higher-order oligomers induced by the ligand.

### The EphA2 L913F mutation promotes receptor dimerization but not the formation of higher-order oligomers

**Figure 5:**
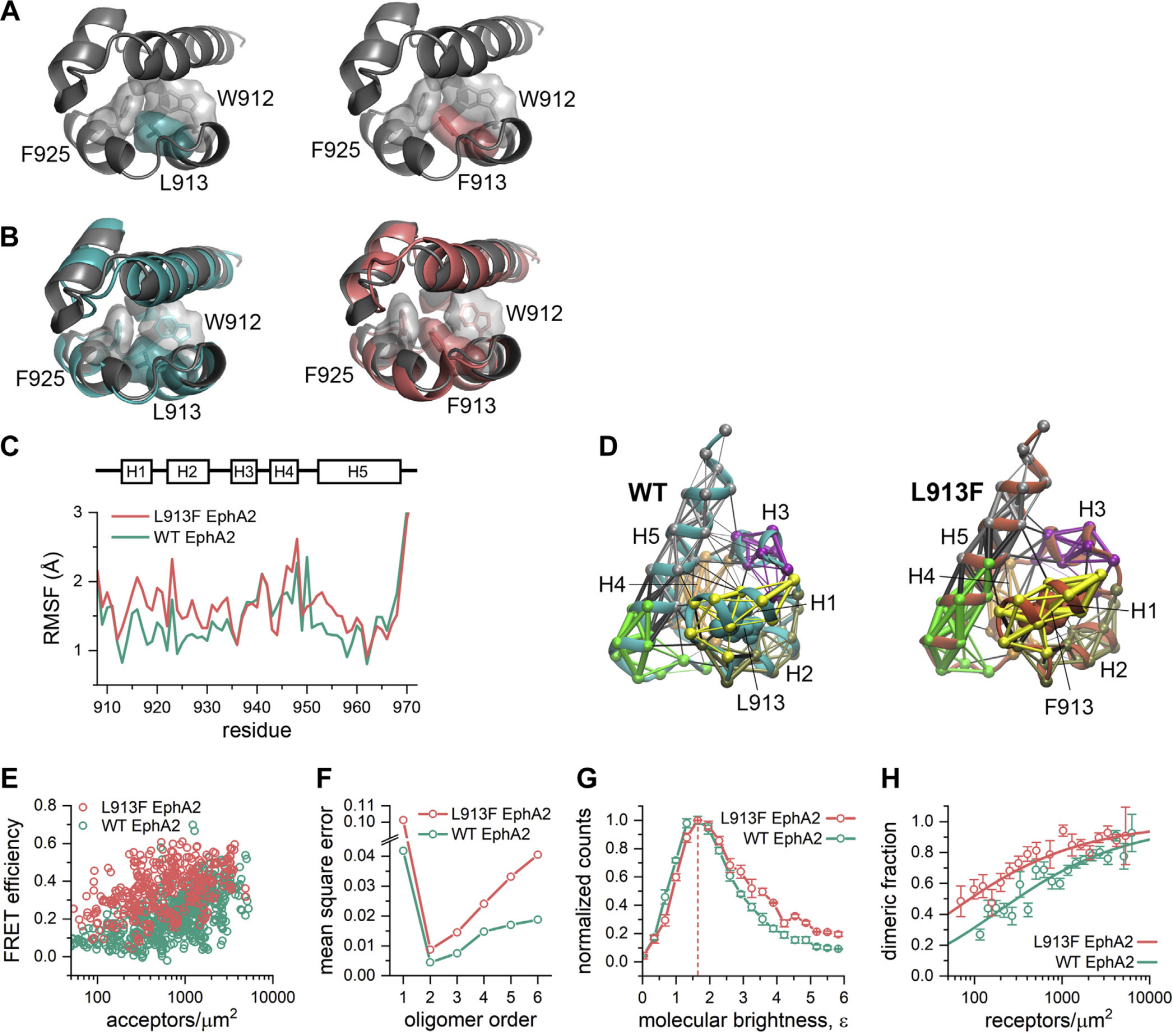
**The EphA2 L913F mutation does not promote the formation of higher-order receptor oligomers.***A*, structure of the EphA2 WT SAM domain (*left*; PDB ID: 2KSO, chain A) and model of the L913F mutant (*right*) in r*ibbon* representation with the indicated residues shown as sticks and as a molecular surface. The model, created through a direct substitution of L913 with F, illustrates how W912 and F925 interfere with a phenylalanine at position 913. *B*, molecular dynamics simulations of the EphA2 WT (*left*) and L913F (*right*) SAM domains are aligned to the EphA2 SAM domain crystal structure (*gray*) by minimizing the backbone RMSD values relative to the crystal structure. *C*, the RMSF values calculated using the crystal structure coordinates as the reference, are plotted for each residue in the EphA2 WT and L913F SAM domains over the course of the MD simulations, each averaged over three replicates. A schematic of the SAM domain helix positions is shown above the figure. *D*, dynamic network analysis of the EphA2 WT and L913F SAM domain structures. Nodes, indicated by *spheres*, highlight the α-carbon atoms and the thickness of the edges (lines connecting the nodes) is proportional to the correlation of atomic motion in space and time. The WT L913 or mutant F913 is rendered with atoms as *spheres*. Six communities are shown in different colors: community 1 in *yellow*, community 2 in *olive*, community 3 in *purple*, community 4 in *orange*, community 5 in *green*, and community 6 in *gray*. Edges drawn within the same community are colored according to that community and edges drawn between nodes of different communities are colored *black*. The α-helices (H1 through H5) are indicated. *E*, comparison of FRET efficiencies measured for the EphA2 WT and L913F mutant in HEK293T cells in the absence of ligand. *F*, MSE values calculated for EphA2 WT and the L913F mutant. *G*, FIF histograms showing the molecular brightness distributions for EphA2 WT and L913F in the absence of ligand. The brightness values corresponding to the histogram maxima are indicated by the *dotted line*. *H*, the dimeric fractions calculated from the FSI-FRET data for EphA2 L913F are compared with those calculated for EphA2 WT. The *symbols* represent the binned and averaged dimeric fractions and are shown with the standard errors. The *solid lines* are the best fit curves for monomer–dimer equilibrium (see Table 1). The data for EphA2 WT in *F*–*H* are from (47) and are shown here for comparison. FRET, Föster resonance energy transfer.

Sequence alignment reveals that EphA4 L920 is conserved in all Eph receptors (29). However, cancer mutations of this particular leucine have not been reported for EphA2 (cbioportal.org), an Eph receptor that is highly expressed in many cancer types (7). To determine whether the consequences of the mutation of this conserved leucine to phenylalanine are similar in other Eph receptors, we characterized the effects of the analogous L913F mutation in EphA2. Similar to the EphA4 L920F mutation, the EphA2 L913F mutation has a PolyPhen-2 score of 1.00 (genetics.bwh.harvard.edu/pph2), indicative of probable functional effects. An EphA2 SAM domain structure (PDB ID: 2KSO, chain A) shows that the side chain of L913 in helix 1 is buried in the protein core in close proximity to W912 in helix 1 and F925 in helix 2 (Fig. 5*A*, left). Thus, the packing of the EphA2 SAM domain core appears similar to EphA4. Just as for EphA4, the molecular surface of F913 clashes with F925, and thus a phenylalanine cannot fit in the available space (Fig. 5*A*, right).

To understand the consequences of the L913F mutation on the dynamics and stability of the EphA2 SAM domain, we performed MD simulations of EphA2 WT and the L913F mutant, starting from the EphA2 SAM domain crystal structure. Comparison of the two MD-derived EphA2 SAM domain structures with the crystal structure shows few variations for both EphA2 WT (Fig. 5*B*, left) and the L913F mutant (Fig. 5*B*, right). However, it should be noted that one of the MD simulation replicates (replicate 3) for the EphA2 L913F mutant SAM domain demonstrated larger conformational variations than the EphA2 WT SAM domain (Fig. S1). Replicates 1 and 2 for the L913F SAM domain show similar RMSD fluctuations as the WT SAM domain. Consistent with this, the RMSF differences between the residues in the EphA2 WT and L913F mutant SAM domains (Fig. 5*C*) are similar to each other and less pronounced than for EphA4 (Fig. 3*C*).

Community clustering based on the MD trajectories of the EphA2 SAM domain shows similar communities as observed for EphA4 (Fig. 5*D*). The EphA2 L913F mutation greatly reduces interhelical coupling, since many of the interhelical edges (black) in the EphA2 WT SAM domain are not present in the EphA2 L913F SAM domain. Similar to the analysis of the EphA4 SAM domains, the L913F mutation also stabilizes helix 1, containing the mutation, along with helices 2, 3, and 5, as shown by the thicker edges within these communities (Fig. 5*D*). We also observed some notable differences between the network analyses of the EphA4 and EphA2 SAM domains. Helix 1, which is largely interconnected with several other communities in the EphA4 WT SAM domain (Fig. 3*D*), is only connected with itself in the EphA2 WT SAM domain (Fig. 5*D*). Thus, stabilization of helix 1 is observed for both the EphA4 L920F and EphA2 L913F SAM domains but is accompanied by a loss of the community coupling connecting helix 1 to other helices only for the EphA4 SAM domain and not for the EphA2 SAM domain. Additionally, helix 4 is an independent community in both the EphA2 WT and L913F SAM domains, which is different for the EphA4 SAM domains. These differences may explain why we observe a structural change for the EphA4 L920F mutant but not for the EphA2 L913F mutant, although further validations are needed.

We measured somewhat higher FRET efficiencies for the EphA2 L913F mutant compared with previously published data for EphA2 WT (47) (Fig. 5*E*). The MSE value calculated for the EphA2 L913F mutant is lowest for the dimer model (*n* = 2), similarly to EphA2 WT (Fig. 5*F*). This suggests that the L913F mutation promotes the formation of EphA2 dimers and not higher-order oligomers, which is different from the effects of the L920F mutation in EphA4. FIF analysis confirmed that the L913F mutation does not affect the brightness distributions, and thus oligomer size, of EphA2 L913F compared with WT (Fig. 5*G*). The dissociation constant calculated from the dimerization curve for EphA2 L913F is 90 ± 20 receptors/μm^2^ (Fig. 5*H* and Table 1), which is lower than the dissociation constant we previously reported for EphA2 WT (300 ± 70 receptors/μm^2^) (47). Thus, the L913F mutation stabilizes EphA2 dimers, promoting their formation at lower receptor concentrations.

### A model for oligomerization of the EphA4 L920F mutant

**Figure 6:**
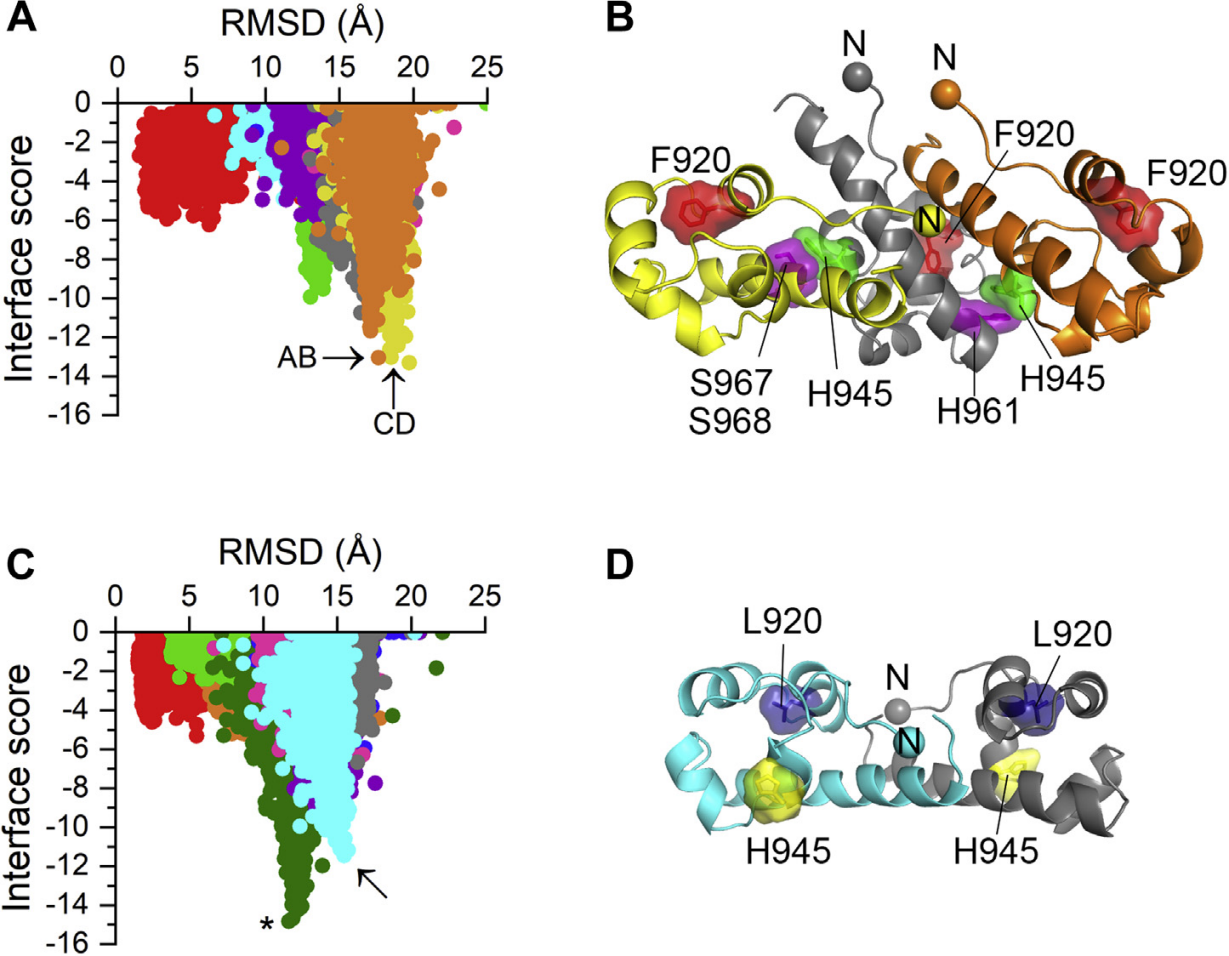
***In silico* docking predicts two stable interfaces for the EphA4 L920F SAM domain.***A*, ten EphA4 L920F SAM dimer structures generated by ClusPro were optimized by creating 2000 decoys for each with PyRosetta. Interface scores for the decoys are plotted as a function of the RMSD value calculated relative to the initial ClusPro structure “0” (ClusPro outputs are numbered 0–9). Each of the ten sets of decoys is shown in a *different color*. The lowest-energy decoys from each set represent the optimized dimer structures. Two lowest-energy dimer structures for the EphA4 L920F SAM domain, referred to as AB (*orange*) and CD (*yellow*), are indicated by *arrows*. The decoy with the lowest interface score for the set of structures colored in *yellow* was considered an outlier (see Fig. S5), so the second lowest was selected. *B*, model of an EphA4 L920F SAM domain trimer that engages both AB and CD interfaces. The three SAM domains in the trimer are shown in *orange* (molecule A), *yellow* (molecule D), and *gray* (molecule B/C). Residue F920 is shown in *red* as *sticks* and as a molecular surface. Residue H945, shown in *green* as sticks and as a molecular surface, stabilizes both interfaces (see Table S1) by engaging H961 (*purple sticks* and molecular surface) in molecule B/C and S967/S968 (*purple sticks* and molecular surface) in molecule D. *C*, ten sets of EphA4 WT decoys were generated as described in *A*. A single lowest-energy structure, indicated by an *asterisk*, was identified for the EphA4 WT SAM domain (*green*). However, this model did not match some experimental data and therefore the second lowest structure (*cyan*), indicated by an *arrow*, was selected for further analysis. *D*, structural representation of the second lowest-energy EphA4 WT dimer structure in which the two SAM domains (A and B) are indicated in *cyan* and *gray*. Residue L920 is indicated in *blue* and residue H945 in *yellow*, both shown as *sticks* and as a molecular surface. This model is structurally similar to the crystallographic dimer shown in Fig. S6*C*.

The FRET, FIF, and immunoblotting data suggest that the EphA4 L920F mutation promotes receptor oligomerization through effects on the SAM domain. To identify possible interfaces between SAM domains harboring the L920F mutation, we generated *in silico* predictions by docking the L920F SAM domain structures from the MD simulations using the ClusPro 2.0 (48, 49) and PyRosetta computational resources (50). Two L920F mutant SAM domains were first docked together using the ClusPro software to create ten low-energy SAM domain dimer arrangements (numbered 0–9). The ten dimer structures were then processed with PyRosetta to further refine the dimer interface. Using PyRosetta, we created 2000 decoys for each of the ten EphA4 SAM dimer structures by introducing randomized perturbations, resulting in ten sets of decoys and 20,000 total structures. We used Rosetta scoring functions to score the interface of each dimer decoy and calculated the RMSD for each decoy using the initial structure “0” from ClusPro as a reference. By plotting the interface scores as a function of the RMSD values, we obtained an “energy funnel” for each set of decoys originating from the same ClusPro structure (Fig. 6*A*). The shapes of these funnels show that dimer decoys with high interface scores (approaching zero and indicating higher-energy interfaces) have a wide range of RMSD values, whereas decoys with low interface scores (indicating lower-energy interfaces) typically converge to single RMSD values. The interface scores for the ten ClusPro dimer models prior to optimization with PyRosetta are all greater than 0 with the exception of an L920F structure that scored −2.23. Therefore, PyRosetta successfully generated lower-energy dimer models from the ClusPro structures.

Decoys with the lowest interface scores were selected as the optimized dimer structures, although low interface score decoys that deviated more than 1 Å from the mean RMSD value were considered as outliers (Fig. S5). This analysis identified two different EphA4 L920F SAM domain dimer structures with similarly low interface scores (dimers AB and CD in Fig. 6*A*), suggesting that there are two possible low-energy dimer structures. The two SAM domains are oriented parallel to each other in dimer AB and nearly perpendicular to each other in dimer CD. We created a model of a SAM domain trimer by aligning SAM domain B from dimer AB with SAM domain C from dimer CD (Fig. 6*B*). We observed no steric clashes upon aligning the two dimer structures to generate the trimer model, indicating that the two dimer structures engage distinct interfaces. The amino acid residues that are engaged in each interface were determined by calculating the distances between residues on the opposing surfaces of an interface, using a distance restraint of 4.0 Å. The predicted interface residues were analyzed by *in silico* alanine scanning mutagenesis, which supported the importance of the residues and identified those predicted to form the most critical contacts (Table S1). This analysis suggests that H945 plays a key role in both of the predicted L920F SAM domain interfaces by making contact with H961 in interface AB and with a pair of serine residues (S967 and S968) in interface CD (Fig. 6*B* and Table S1).

For comparison, we carried out a similar ClusPro/PyRosetta analysis for the EphA4 WT SAM domain, using the structure obtained from the MD simulations as a starting point. The PyRosetta interface scores identified a single most favorable dimer structure for the WT SAM domain (Fig. 6*C*, asterisk). While this structure (Fig. S6*A*) is predicted to be the most energetically favorable, mutagenesis of a predicted interface residue (N963K) had no effect on EphA4 dimerization measured by FRET (not shown). Therefore, we considered instead the second lowest-energy dimer structure (Fig. 6*C*, arrow). This WT dimer model (Fig. 6*D*) resembles a crystallographic EphA4 SAM dimer structure (Fig. S6, *B* and *C*) that has been experimentally verified by mutagenesis in the context of the isolated SAM domain (30). Of note, the N963 residue is not located near the interface in this model.

**Figure 7:**
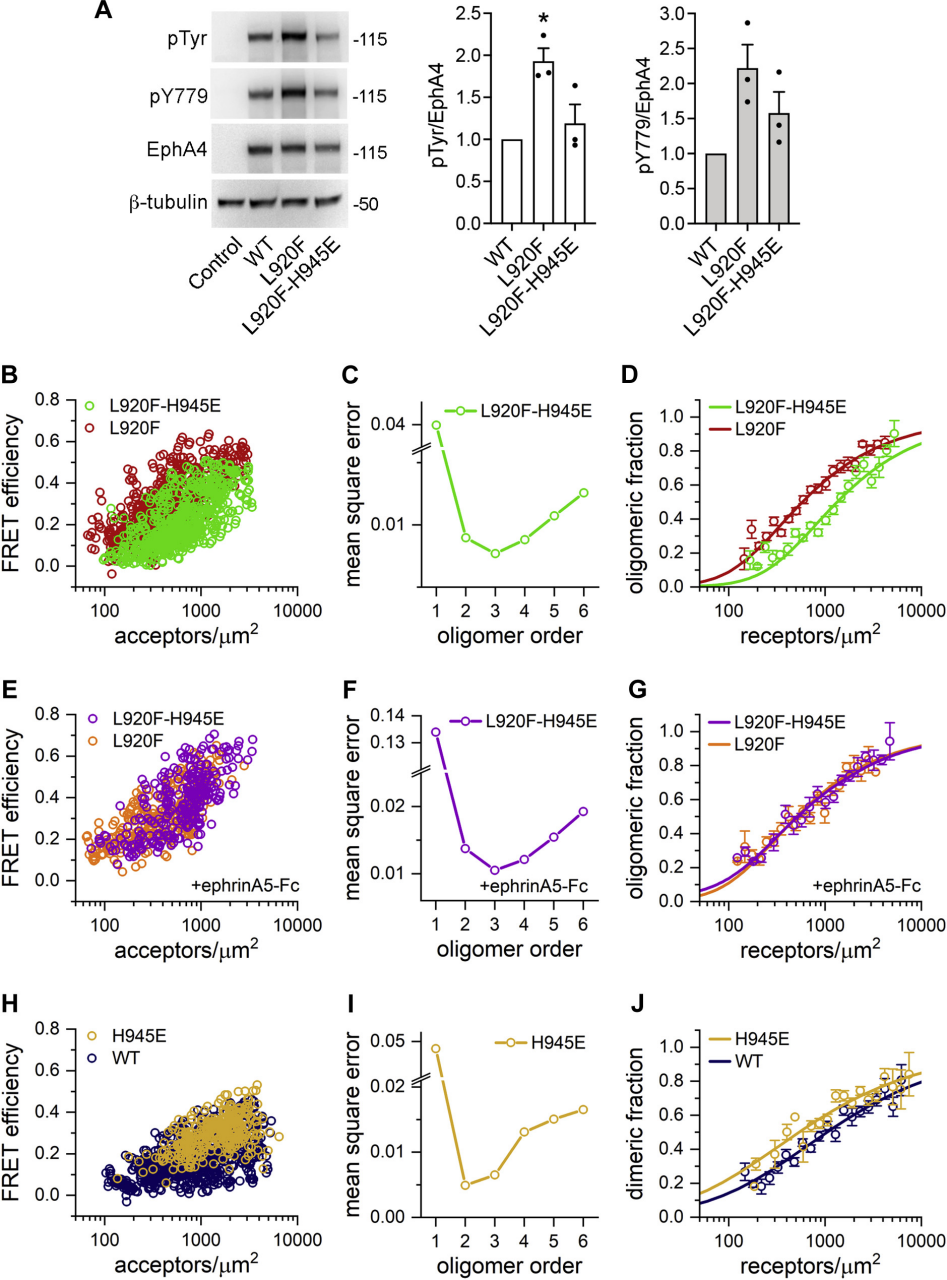
**The H945E mutation destabilizes EphA4 L920F trimers.***A*, HEK293 cells were transiently transfected with constructs encoding EphA4 WT, the L920F single mutant, the L920F-H945E double mutant or EGFP as a control. Cell lysates were probed by immunoblotting with antibodies recognizing phosphotyrosine (pTyr), the Y779 phosphorylated motif (pY779), and EphA4. The bar graph shows averages and standard errors from quantifications of three experiments (individual values from each experiment are shown as *dots*). ∗*p* < 0.05 for the comparison with WT = 1 by one-sample *t* test. *B*, FRET efficiencies measured for the EphA4 L920F-H945E double mutant in HEK293T cells and compared with those for the L920F mutant. *C*, MSE values calculated for EphA4 L920F-H945E. *D*, oligomeric fractions calculated from the FSI-FRET data are plotted as a function of total receptor concentration for the EphA4 L920F-H945E double mutant compared with the L920F mutant (see Table 1). *E*, FRET efficiencies measured for EphA4 L920F-H945E in the presence of ephrinA5-Fc as a function of acceptor concentration and compared with the EphA4 L920F mutant. *F*, MSE values for EphA4 L920F-H945E in the presence of ligand. *G*, the oligomerization curve calculated from the FSI-FRET data for EphA4 L920F-H945E in the presence of ephrinA5-Fc compared with that for EphA4 L920F (see Table 1). *H*, FRET efficiencies measured for the EphA4 H945E mutant as a function of acceptor concentration and compared with EphA4 WT. *I*, MSE values calculated for EphA4 H945E. *J*, the oligomerization curve calculated from the FSI-FRET data for the EphA4 H945E mutant and compared with EphA4 WT (see Table 1). FRET, Föster resonance energy transfer.

Based on these models, we hypothesized that mutation of H945 should selectively destabilize EphA4 L920F oligomers. An EphA4 L920F-H945E double mutant indeed exhibited decreased tyrosine phosphorylation compared with the L920F single mutant (Fig. 7*A*), supporting our model. We also detected a decrease in the FRET efficiencies of the EphA4 L920F-H945E double mutant compared with the L920F single mutant (Fig. 7*B*). An MSE value minimized for *n* = 3 indicates that the EphA4 L920F-H945E double mutant forms trimers (Fig. 7*C*), as in the case for EphA4 L920F (Fig. 4*D*). Comparing the oligomerization curves for the EphA4 L920F-H945E and L920F mutants shows that the H945E mutation destabilizes EphA4 L920F oligomers (Fig. 7*D*), consistent with the ClusPro/PyRosetta predictions (Table S1). The oligomeric fraction for the EphA4 L920F-H945E double mutant is ∼50% at concentrations of ∼1350 receptors/μm^2^, which is substantially higher than the ∼580 receptors/μm^2^ for EphA4 L920F (Table 1). Thus, the FRET data show that the H945E mutation decreases the oligomerization propensity of the L920F mutant by destabilizing but not completely disrupting the AB and/or CD interfaces.

We observed greater FRET efficiencies for L920F-H945E in the presence of ephrinA5-Fc than in the absence of ligand (Fig. S4*G*), which suggests that ephrinA5-Fc stabilizes EphA4 L920F-H945E oligomers. Indeed, ephrinA5-Fc shifted the oligomerization curve to lower receptor concentrations compared with the absence of ligand (Fig. S4*H*). In the presence of ephrinA5-Fc, the FRET efficiencies measured for EphA4 L920F-H945E are very similar to the FRET efficiencies for EphA4 L920F (Fig. 7*E*). Furthermore, MSE analysis shows that EphA4 L920F-H945E still forms trimers (MSE minimum at *n* = 3) in the presence of ephrinA5-Fc (Fig. 7*F*), and the oligomerization curves for EphA4 L920F-H945E and EphA4 L920F in the presence of ligand are very similar (Fig. 7*G*). From the oligomerization curve, we estimated the apparent dissociation constant to be ∼530 receptors/μm^2^ for EphA4 L920F-H945E in the presence of ephrinA5-Fc (Table 1). Thus, the H945E mutation destabilizes the EphA4 L920F oligomers in the absence but not in the presence of ligand. This is consistent with the idea that ligand binding can induce structural changes in RTK oligomers (51, 52, 53, 54).

The H945 residue is not predicted to destabilize the WT dimer interfaces (not shown), and thus we engineered an EphA4 H945E single mutant to verify this prediction using FSI-FRET. We found that the H945E mutation slightly increases the FRET efficiency compared with EphA4 WT (Fig. 7*H*). The MSE value is minimized for an oligomer order of *n* = 2 (Fig. 7*I*), indicating that EphA4 H945E associates into dimers similar to EphA4 WT (Fig. 4*D*). The calculated dimeric fraction suggests that the H945E mutation slightly stabilizes EphA4 dimers compared with WT (Fig. 7*J*). The dissociation constant of 540 ± 120 receptors/μm^2^ for EphA4 H945E is smaller than for EphA4 WT (1050 ± 170 receptors/μm^2^; Table 1). Therefore, in the absence of ligand, the H945E mutation destabilizes EphA4 L920F oligomers but stabilizes EphA4 WT dimers, supporting the notion that the SAM domain functions differently in EphA4 WT and the L920F mutant.

## Discussion

Eph receptors are known to form dimers and larger oligomers in response to the binding of ephrin ligands, leading to cross-phosphorylation of Eph receptor molecules on tyrosine residues, increased kinase activity, and downstream signaling (6, 7, 26). Multiple domains in the Eph receptor extracellular and intracellular regions have been implicated in receptor–receptor association at the plasma membrane (6, 55). Interestingly, the role of the SAM domain in Eph receptor oligomerization seems to vary in different Eph receptors, since SAM domain deletion inhibits EphA3 oligomerization but promotes EphA2 oligomerization and signaling (35, 42, 43). In the case of EphA4, deletion of the SAM domain did not detectably affect receptor tyrosine phosphorylation as well as signaling in *Xenopus* embryos and the developing mouse corticospinal tract (56, 57). Our FRET data corroborate this finding since deletion of the SAM domain in EphA4 ΔSAM does not alter EphA4 dimerization propensity. Thus, the SAM domain has remarkably diverse effects on the assembly of these closely related Eph receptors in the plasma membrane.

Here we show that the EphA4 L920F melanoma mutation, which is located in the SAM domain, induces dysregulated receptor autophosphorylation and signaling. Therefore, this mutation causes aberrant EphA4 activation, which may lead to pathological consequences. The L920 residue is also mutated (to proline) in a uterine endometrial carcinoma tumor, and the L920P mutation is also predicted to have a strong impact on EphA4 function (cbioportal.org). It will therefore be interesting in the future to determine whether the L920P mutation also promotes EphA4 activation. Interestingly, the engineered Y928F mutation in the EphA4 SAM domain has also been reported to increase EphA4 signaling, possibly by inhibiting SAM domain phosphorylation (57).

Previous studies have suggested that EphA4 activation in different tumor cell types can affect cancer progression. Both tumor-promoting and tumor-suppressing activities have been reported for EphA4 (9, 10, 11, 12, 13, 14, 15, 16, 21), suggesting that the consequences of EphA4 activation in cancer cells may vary, for example, depending on the cellular context. In agreement with a dual role of EphA4 even in the same tumor type, our analysis of EphA4 melanoma mutations identified two mutations (G733W and R745C) that abrogate EphA4 kinase activity and the L920F mutation, which increases kinase activity. To our knowledge, G733W, R745C, and L920F are the first cancer mutations reported to affect EphA4 function. We focused on the L920F gain-of-function mutation because patient survival analyses suggest a correlation between EphA4 activation and melanoma malignancy.

Since the L920F mutation is located in the SAM domain, the observed increase in kinase activity is not due to direct effects of the mutation on the kinase domain. Instead, we found that the mutation promotes the interaction between EphA4 molecules in the plasma membrane. Our immunoblotting and FRET data show that the L920F mutation enhances EphA4 oligomerization and activation in the absence of ligand. Thus, the strong ligand-independent activation of the EphA4 L920F mutant is a direct consequence of SAM domain-mediated EphA4 oligomerization. Interestingly, while EphA4 WT forms dimers at high receptor densities, our FRET and FIF data along with the docking simulations highlight the propensity of EphA4 L920F to form trimers. Docking simulations suggest that the EphA4 L920F trimers assemble using two SAM domain interfaces that differ from the single predicted interface mediating EphA4 WT dimers. Differences in the SAM domain interfaces engaged may lead to different signaling properties.

The predicted EphA4 WT dimer configuration is in good agreement with the dimer configuration observed in the crystal structure of the EphA4 SAM domain (30). However, the EphA4 WT SAM-SAM interface does not seem to play a critical role in receptor assembly on the cell surface, since our FRET data for EphA4 WT and ΔSAM are very similar (Fig. S3, *A*–*C*). This could be due to a weak interface between the EphA4 WT SAM domains, which has a negligible contribution compared with receptor–receptor interfaces in other EphA4 regions. Alternatively, the EphA4 WT SAM domain interface shown in Figure 6*D* may play a stabilizing role (as reported for EphA3 (35)), but this role is counterbalanced by destabilizing effects (as reported for EphA2 and EphB2 (42, 43, 58)) that may occur indirectly, perhaps involving the kinase domain. On the other hand, comparison of FRET data for EphA4 L920F and EphA4 ΔSAM (Fig. S3, *D* and *E*) highlights the critical role of the L920F mutant SAM domain, which is responsible for both altering the oligomeric state of EphA4 and increasing the EphA4 oligomeric fraction in the absence of ligand.

Pathogenic single amino acid mutations in receptor tyrosine kinases are often found in interfaces that form between receptor molecules in dimers or higher-order oligomers (59, 60). L920 in EphA4, however, is buried in the core of the SAM domain. Although the mutant F920 is predicted to be solvent exposed, neither L920 in EphA4 WT nor F920 in the EphA4 mutant appears to directly participate in any of the low-energy EphA4 SAM domain interfaces identified in our MD simulations. Rather, the substitution of L920 with phenylalanine affects the fold of the SAM domain, and the induced structural changes lead to the engagement of new SAM domain interfaces that mediate the formation of SAM domain trimers.

While ligands are generally believed to be required for oligomerization and activation, recent work has established that many receptor tyrosine kinases (such as members of the EGF, FGF, and VEGF receptor families and others) form dimers even in the absence of ligand and that these dimers possess kinase activity and autophosphorylate (51, 52, 54, 61, 62, 63). The Eph receptors are no exception in this regard. Both EphA2 and EphA3 can form dimers in the absence of ligand (34, 35), and here we show that EphA4 does so as well. EphA4 activating mutations, such as L920F, could promote malignancy in melanomas in which ligand-dependent EphA4 activation is low. Unliganded receptor tyrosine kinase dimers with increased kinase activity are known to play a role in disease, particularly in tumors, where receptor tyrosine kinases are often overexpressed (64, 65, 66, 67). Here we show that an EphA4 mutation profoundly affects receptor–receptor interaction and alters the oligomer size in the absence of ligand binding, further highlighting the role of unliganded receptor tyrosine kinase oligomeric assembly in disease.

We also show that the EphA4 L920F mutant can still be activated by the ephrinA5 ligand if its expression is low. Although this effect cannot be explained by the FRET analyses, our FIF analyses show that ephrinA5-Fc induces the formation of larger oligomers for both EphA4 WT and L920F. Therefore, at the low receptor concentration characterizing the stably transfected cells used in the immunoblotting experiments shown in Figure 2*C*, the higher-order oligomers induced by ephrinA5-Fc can increase EphA4 L920F activity. Interestingly, mutation of H945 in the L920F-H945E double mutant destabilizes the EphA4 L920F mutant in the absence of ligand but not when the cells are treated with ephrinA5. Thus, it is conceivable that ephrin ligand binding induces structural changes in the EphA4 trimers that propagate from the extracellular region to the SAM domain without affecting the oligomerization state of EphA4. This has been proposed for other families of receptor tyrosine kinases known to function as dimers, such as the IGF-1, EGF, FGF, VEGF, and Trk receptor families (51, 52, 54, 68, 69). In addition, ligand-mediated interactions in the EphA4 extracellular region must work synergistically with SAM-SAM interactions to stabilize oligomeric assemblies of highly phosphorylated EphA4 L920F molecules. Interestingly, previous crystal structures of the EphA4 extracellular region in complex with ephrin ligands also revealed the formation of trimeric or hexameric structures (27). Thus, the EphA4 extracellular region and the mutant SAM domain both appear to have the propensity to form trimeric assemblies.

Although EphA4 L920 is highly conserved in all Eph receptors including EphA2, we did not detect higher-order oligomerization of the corresponding EphA2 L913F mutant. Rather, we found that the EphA2 L913F mutation increases EphA2 dimerization. This may be explained at least in part by the fact that EphA4 H945, which is important for both of the predicted interfaces in the EphA4 L920F SAM domain trimer, is not conserved in other Eph receptors including EphA2, where it is a glutamine. It can be speculated that a change in oligomer order, from a dimer to a trimer (as observed here for the EphA4 L920F mutant and previously for engineered EphB2 mutants (58)), may have a more significant effect on signaling and pathological effects compared with dimer stabilization (as observed for the EphA2 L913F mutant).

There are many examples of single amino acid mutations in receptor tyrosine kinases that have been implicated in human pathologies. Studies over the past 2 decades have shown that such mutations can affect receptor function *via* many diverse mechanisms. Examples include receptor dimer stabilization (60), enhancement in ligand-binding affinity (70, 71), and structural changes in receptor dimers (51). Here we uncover a fundamentally novel mechanism through which a single amino acid mutation in a receptor tyrosine kinase can cause disease by altering the size of the signaling oligomers. Furthermore, our findings suggest a pathogenic role for EphA4 kinase-dependent signaling in melanoma, implying that EphA4 kinase inhibitors may have therapeutic utility.

## Experimental procedures

### DNA constructs

Human EphA4 cDNA (GenBank accession number NM_001304536.2) was cloned into the pLVX-IRES-Neo lentivirus with an N-terminal FLAG tag (DYKDDDDK) or Strep tag (WSHPQFEK) sequence. The EphA4 WT sequence was also cloned into pcDNA3 with a C-terminal sequence encoding a (GGS)_5_ linker followed by EYFP or mTURQ. The FLAG-EphA4 L920F mutant was generated by overlapping PCR, and a restriction fragment containing the mutation was subcloned to generate the Strep-, mTURQ-, and EYFP-tagged EphA4 L920F constructs. The mTURQ- and EYFP-tagged EphA4 H945E and EphA4 L920F-H945E mutants were generated by overlapping PCR with the QuikChange II site-directed mutagenesis kit (Agilent Technologies) using the respective pcDNA3-EphA4-mTURQ or pcDNA3-EphA4-EYFP as the templates. The mTURQ- and EYFP-tagged EphA4 ΔSAM mutant (lacking amino acids 910–983, corresponding to the SAM domain) was generated by overlapping PCR. The human EphA2-EYFP WT and EphA2-mTURQ WT constructs (GenBank accession number NM_004431.5) in pcDNA3 used for FRET have been previously described (47). N-terminally FLAG-tagged EphA2 wild-type in the pLVX-IRES-Neo lentiviral construct and the pLVX-IRES-Neo-EGFP control construct have also been described (72). The pLVX-IRES-Neo-FLAG-EphA2 L913F mutant was generated by overlapping PCR, and a restriction fragment containing the mutation was subcloned to generate the mTURQ- and EYFP-tagged EphA2 L913F constructs.

### Cell culture and transfection

HEK293AD human embryonic kidney cells (Cell Biolabs, AD-100) were transiently or stably transfected for pull-downs, immunoprecipitations, and immunoblotting. The cells were cultured in Dulbecco’s modified eagle medium (DMEM; Corning, 10-013-CV) containing 10% fetal bovine serum as well as antimycotics and antibiotics (Corning, 30-004-Cl) and transfected using Lipofectamine 2000 reagent according to the manufacturer’s recommendations (Thermo Fisher Scientific/Invitrogen). Transiently transfected cells were used 48 h after transfection or selected with 1 mg/ml G418 (Thermo Fisher Scientific) for 15 days to generate stably transfected cells.

HEK293T cells (American Type Culture Collection) were used for FRET experiments. The cells were seeded in 35 mm glass bottom collagen-coated dishes (MatTek Corporation) and cultured overnight at 37 °C in 5% CO_2_ using DMEM supplemented with 10% fetal bovine serum, 3.5 g/l D-glucose, and 1.5 g/l sodium bicarbonate. The cells were then transiently cotransfected with pcDNA3-EphA4-mTURQ and pcDNA3-EphA4-EYFP WT, ΔSAM mutant, L920F mutant, L920F-H945E mutant, or H945E mutant (1–5 μg total DNA) using the manufacturer’s recommendations for the Lipofectamine 3000 reagent (Thermo Fisher Scientific/Invitrogen). The cells were also similarly cotransfected with pcDNA3-EphA2 L913F-mTURQ and pcDNA3-EphA2 L913F-EYFP (1–2 μg total DNA). Twelve hours following the transfection, the cells were rinsed twice with starvation medium to remove traces of phenol red and serum-starved overnight to ensure no soluble ligands were present. Immediately before imaging, the starvation medium was replaced with hypo-osmotic medium (10% starvation medium, 90% water, 25 mM HEPES) to reversibly “unwrinkle” the cell membrane as described (73). Cells were imaged under these conditions for approximately an hour. In some cases, ephrinA5-Fc ligand (R&D Systems, 374-EA-200) was added to the hypo-osmotic medium to a final concentration of 0.5 μg/ml and premixed, prior to adding it to the imaging dishes. All surfaces were pretreated with 7.5% BSA in phosphate buffered saline (PBS) to prevent loss of ligand due to surface adsorption.

### Cell lysates, Strep-Tactin pull-downs, and immunoprecipitations

Cells were rinsed once with ice-cold PBS containing Ca^+^ and Mg^+^ (Lonza, 17-513F) and collected in sodium dodecyl sulfate sample buffer or Bolt lithium dodecyl sulfate (LDS) sample buffer (Life technologies, B0007) with 2.5% β-mercaptoethanol and Halt Protease and Phosphatase inhibitor cocktail (Fisher Scientific, 78443). Lysates were heated at 95° for 2 min and briefly sonicated.

For Strep-Tactin pulldowns, cells were cultured until they reached ∼80% confluency, rinsed with ice-cold PBS containing Ca^+^ and Mg^+^, and collected in PBS buffer containing 0.5% Triton X-100 and Halt Protease and Phosphatase inhibitor cocktail (Fisher Scientific, 78443). Cells were incubated for 5 min on ice with periodic mixing and centrifuged for 10 min at 16,700*g* at 4 °C to remove insoluble material. The supernatants were further precleared by incubation with Sepharose beads (Sigma-Aldrich, 4B200) for 15 min at 4 °C on a rotator followed by centrifugation. Each pull-down was performed by incubating 20 to 25 μl of Strep-TactinXT Superflow resin beads (IBA, 2-4010-010) with cell lysates for 2 h at 4 °C on a rotator. The immunoprecipitates were washed four times with 1 ml PBS with 0.5% TX-100 and once with PBS and eluted by incubation at 95 °C for 2 min in 25 μl Bolt LDS sample buffer with 2.5% β-mercaptoethanol.

For immunoprecipitations, cells were cultured until they reached ∼80% confluency and lysed. Alternatively, for ephrinA5-Fc stimulation, cells were rinsed with prewarmed PBS containing Ca^+^ and Mg^+^ and serum-starved for 2 h, incubated for 10 min at 37 °C with 0.5 μg/ml ephrinA5-Fc (R&D Systems, 374-EA-200) or human Fc (MP Biomedicals, #55911) as a control, and then washed with ice-cold PBS containing Ca^+^ and Mg^+^. Cells were lysed in modifed RIPA buffer (1% Triton X-100, 0.5% sodium deoxycholate, 0.1% sodium dodecyl sulfate, and 2 mM EDTA in PBS, pH 7.5) containing Halt Protease and Phosphatase inhibitor cocktail by incubation for 5 min on ice with periodic mixing. Cell lysates were centrifuged for 10 min at 16,700*g* at 4 °C to remove insoluble material. The supernatant was further precleared by incubation with Sepharose beads for 15 min at 4 °C on a rotator followed by centrifugation. Each FLAG immunoprecipitation was performed by incubating 20 to 25 μl of anti-FLAG M2 affinity gel (Sigma-Aldrich, A2220) with cell lysates for 2 h at 4 °C on a rotator. The immunoprecipitates were washed three times with 1 ml PBS with 0.5% TX-100 and once with PBS and eluted by incubation at 95 °C for 2 min in 25 μl Bolt LDS-containing sample buffer without β-mercaptoethanol (to avoid dissociating the chains of the FLAG antibody not directly linked to the beads). Following centrifugation for 1 min at 1000*g*, the supernatant was collected and β-mercaptoethanol was added to a final concentration of 2.5%.

### Immunoblotting

Lysates, pull-downs, and immunoprecipitations were run on Bolt 4 to 12% Bis-Tris Plus gels (Invitrogen, NW04125). After semidry transfer, the immobilon membranes were blocked with 5% bovine serum albumin in 0.1% Tween-20 in TBS (150 mM NaCl, 50 mM TrisHCl pH 7.5) for 1 h and then incubated overnight at 4 °C in blocking buffer containing primary antibodies recognizing EphA4 (BD Biosciences, 610471; 1:1000; Figs. 1*D*, 2, *A*–*C*, 4*B* and 7*A*, an affinity-purified rabbit polyclonal EphA4 antibody generated using a peptide corresponding to the 11 C-terminal amino acids of human and mouse EphA4 (74) and used at 1 μg/ml in Figs. 2*D* and 4*A*); EphA3 pY779 (Cell Signaling Technology, 8862; 1:1000 dilution), which also recognizes EphA4 pY779; β-tubulin (Cell Signaling Technology, 2128; 1:25,000 dilution); the FLAG tag (Sigma, F1804; 1:1000 dilution) and an HRP-conjugated antibody recognizing phosphotyrosine (Cell Signaling Technology, 5465; 1:2000). The Strep tag was detected by incubating the blots overnight with Strep-Tactin-HRP conjugate (IBA GmbH, 2-1502-001; 1:1000 dilution). After overnight incubation, the membranes were washed three times and then incubated at room temperature for 1 h with a horseradish peroxidase (HRP)-conjugated anti-rabbit secondary antibody (Invitrogen, A16110; 1:4000 dilution) or HRP-conjugated anti-mouse secondary antibody (Invitrogen, A16078; 1:4000 dilution) followed by ECL (GE Healthcare, RPN2106) or SuperSignal West Dura (Thermo Fisher, 34076) chemiluminescence detection. The chemiluminescence signal was captured using the ChemiDoc Touch Imaging System (Bio-Rad), quantified using Image Lab (Bio-Rad), and analyzed using Prism software (GraphPad).

### Fully quantified spectral imaging (FSI) FRET imaging

FSI-FRET experiments were carried out following previously published protocols (34, 75, 76). Spectral images of cells under reversible hypo-osmotic conditions were obtained with a spectrally resolved two-photon microscope equipped with the OptiMis True Line Spectral Imaging system (Aurora Spectral Technologies) (77, 78). Fluorophores were excited by a mode-locked laser (MaiTai, Spectra-Physics) that generates femtosecond pulses between wavelengths of 690 nm and 1040 nm. Two images were collected for each cell. A FRET scan was performed at 840 nm to mainly excite the donor fluorophore (mTurquoise), and a second scan was performed at 960 nm to mainly excite the acceptor fluorophore (EYFP). Only regions of the cell membrane not in contact with neighboring cells were imaged, to prevent interactions with ephrin ligands that may be present on neighboring cells. Solutions of purified soluble fluorophores, produced according to published protocols (79), were also imaged at several known concentrations at each of these excitation wavelengths to generate a linear fit, converting pixel-level fluorescence intensities into receptor concentrations (76). This calibration curve together with the two cell image scans was used to calculate the FRET efficiency and the concentration of donor- and acceptor-tagged receptors present in the micron-sized cell membrane regions imaged (76).

Receptor concentrations can only be determined if the topology of the imaged region of the plasma membrane is known. This presents a significant challenge, since the plasma membrane of cells is highly “wrinkled” because it contains microscopic invaginations and protrusions (73). Therefore, we subject the cells to hypo-osmotic conditions to “unwrinkle” the plasma membrane. This process does not induce irreversible cellular damage and is completely reversible in our experiments (80). Furthermore, the FRET efficiencies measured for membrane receptors are not altered by hypo-osmotic conditions (76). The FRET efficiencies and two-dimensional receptor concentrations measured under hypo-osmotic conditions are used to generate binding (dimerization/oligomerization) curves, as described in detail previously (76).

The measured FRET efficiencies are corrected for proximity FRET as described previously (44). For dimers, the FRET efficiency depends on the fraction of dimeric receptors, *f*_*D*_, and on the acceptor fraction, *x*_*A*_, according to:

(1) $$FRET\;=\;f_{D}x_{A}\tilde{E}$$

The intrinsic FRET ($\tilde{E}$) is a structural parameter that depends on the distance between the two fluorophores in the dimer and their relative orientation. The following equation (where R*_total_* is the total receptor concentration, including donors and acceptors) is used to determine the two unknowns *K*_*diss*_ and $\tilde{E}$ (76):

(2) $${\frac{FRET}{x_{A}}}_{\;}=\frac{1}{\left[R_{total}\right]}(\left[R_{total}\right]-\frac{K_{diss}}{4}\left(\sqrt{1+8\left[R_{total}\right]/K_{diss}}-1\right)\tilde{\text{E}}$$

The dimer stability is related to the dissociation constant *K*_*diss*_ = 1/*K* according to:

(3) $$\Delta G\;=\;RT\;\ln\;K_{diss}/10^{6}$$

where *K*_*diss*_ is in units of receptors/μm^2^, with the standard state defined as $K_{diss}^{0}=1$ receptor/nm^2^.

The FRET efficiency for trimers depends on the fraction of trimers, *f*_*trimer*_, and the donor fraction, *x*_*D*_, written as:

(4) $$FRET_{trimer}=\frac{f_{trimer}}{3x_{D}}E$$

E is a parameter (derived in (44)) that encompasses the intrinsic FRET ( $\tilde{E}$), the donor fraction (*x*_*D*_), and the acceptor fraction (*x*_*A*_). The following equation is used to determine the two unknowns *K*_*diss*_ and $\tilde{E}$:

(5) $$FRET_{trimer}=\frac{{\left[M\right]}^{3}}{x_{D}K_{diss}\left[R_{total}\right]}E$$

where the monomer concentration [M] is determined by a root-finding algorithm.

The mean square errors (MSE), calculated as described (50), report on how closely the experimental data compare with the theoretical model. Higher-order oligomer models and calculation of their MSE values have been described in detail (44).

### Fluorescence intensity fluctuations (FIF) measurements and analysis

Images of the basolateral membranes of the cells were acquired in a TCS SP8 confocal microscope (Leica) using the photon counting capabilities of the HyD hybrid detector. The measurements were performed with a 488 nm excitation diode laser and a scanning speed of 20 Hz. The pixel depth was at 12 bits and the image size at 1024 × 1024. The emission spectra of YFP were collected from 520 to 580 nm.

Images were analyzed using the FIF software described in (46). The software performed segmentation of the portion of the basolateral membrane outlined by the researcher, into 15 × 15 pixel regions of interest. After segmentation, data were analyzed using the brightness and concentration calculator in the FIF software (46). For molecular brightness calculations, the following equation was used:

(6) $$\varepsilon =\frac{\sigma^{2}-\sigma_{D}^{2}}{<I>}$$

where $\sigma^{2}$ is the variance of fluorescence across segments, $\sigma_{D}^{2}$ is the variance of the noise of the detector, and <I> is the average fluorescence intensity. For a photon-counting detector, the brightness is (81):

(7) $$\varepsilon =\frac{\sigma^{2}}{<I>}-1$$

The brightness values, calculated for thousands of regions of interest, are potted as histograms.

### Molecular dynamics simulations

Two EphA4 structures were used as the starting structures for MD simulations: (1) the structure of the EphA4 WT SAM domain (PDB ID: 1B0X) and (2) the structure of the EphA4 SAM domain with the L920F mutation, in which L920 was mutated to F920 using the VMD Mutator plugin (82). In addition, two EphA2 structures were generated for MD simulations: (1) the structure of the EphA2 WT SAM domain (PDB ID: 2KSO, chain A) and (2) the structure of the EphA2 SAM domain with the L913F mutation, in which L913 (corresponding to L920 in EphA4) was mutated to F913 also using the VMD Mutator plugin. The structures were solvated using the webserver CHARMM-GUI (83). The proteins are charge-neutral and therefore no ions were added. The geometry of the solvated systems was cubic boxes with each dimension equal to 60 Å.

All simulations were performed using the NAMD2 software (84). Proteins were modeled with the CHARMM36m force field (85) and water molecules with the TIP3P model (86). Short-range electrostatics and van der Waals interactions were set with a cutoff of 12 Å, with switching starting at 10 Å. Long-range electrostatic interactions were modeled using the Particle Mesh Ewald method with 1 Å grid spacing (87). The SETTLE algorithm was used to restrain the hydrogen atom bond length (88). The temperature was controlled at 303 K, and the pressure was controlled at 1 atm by Langevin dynamics (89). For initial equilibration, the integration step was set at 1 fs, and the system was minimized for 5000 steps, then run for 50,000 steps. For the production run, the integration step was set at 2 fs. Three replicates were made for each of the four structures and simulated for 300 ns. The initial 50 ns trajectories were discarded in all analyses. For visualization and docking, each representative MD structure was extracted from the last frame of one randomly chosen replicate. Solvent accessible surface area (SASA) was calculated with the measure command in VMD for the selected residues, with a probe of 1.4 Å radius and a time step size of 1 ns.

Dynamical networks were constructed and visualized using the NetworkView plugin in VMD (90). The last 250 ns of each replicate was analyzed at a time step of 50 ps, yielding a total of 15,000 frames. The α-carbon atoms were selected as the nodes in the network. Edges were constructed for two nodes if the nodes stayed within 4.5 Å from each other for at least 75% of the trajectory and were not from bonded (consecutive) residues. The weight of an edge is proportional to the time-averaged correlation in motion between two nodes (90). Clustering of the nodes and edges into communities was performed using the Girvan–Newman algorithm (91).

### Interface predictions with ClusPro and PyRosetta

The monomeric EphA4 SAM domain structures from the MD simulations were docked using the ClusPro 2.0 software (48, 49). Two EphA4 WT or L920F SAM domains were docked together to generate dimer structure predictions. The top ten dimer structures for EphA4 WT or L920F were selected based on ClusPro ‘VdW+Elec’ (van der Waals and electrostatic) energy calculations. Each dimer interface was further optimized by introducing randomized structural perturbations to generate 2000 structural decoys using a custom-written code that employs the PyRosetta modeling suite (50). The resulting 20,000 dimer structures generated for EphA4 WT and L920F were scored using PyRosetta interface scoring functions (Rosetta Energy Function 2015, REF15) (92). The RMSD for the EphA4 WT and L920F SAM dimers was calculated relative to a preoptimized ClusPro WT and L920F dimer structure, respectively. The dimer structures with the lowest interface score for each set of decoys were selected as the optimized dimer structures. The interface residues for each dimer structure were determined by calculating the distance between amino acid residues on opposing chains in the dimer, using a distance cutoff of 4.0 Å. *In silico* alanine scan mutagenesis was performed on all identified interface residues. For this, interface residues were independently mutated to alanine and the mutated dimer structures were rescored using PyRosetta interface score functions. The change in interface score was determined for each mutant relative to the respective starting structure.

## Data availability

All data generated during these studies are included in the text, figures, and tables of this article and electronic supporting information. Source data or materials will be supplied by the corresponding authors in response to reasonable requests.

## Supporting information

This article contains supporting information (30).

## Conflict of interest

The authors declare that they have no conflicts of interest with the contents of this article.
