# Supplementary material for: A cancer mutation promotes EphA4 oligomerization and signaling by altering the conformation of the SAM domain
Source: J Biol Chem. 2021 Jun 15;297(1):100876. doi: 10.1016/j.jbc.2021.100876 (PMC8260879; doi:10.1016/j.jbc.2021.100876)
Supplement: Supplemental Figures S1–S6 and Table S1 [file mmc1.pdf]

**SUPPORTING INFORMATION**
Supporting figure legends
Supporting Table S1
Supporting Figures S1 to S6

# A Cancer Mutation Promotes EphA4 Oligomerization and Signaling by Altering the Conformation of the SAM Domain

Taylor P. Light[1,5], Maricel Gomez-Soler[2,5], Zichen Wang[3], Kelly Karl[4], Elmer Zapata-Mercado[4], Marina P. Gehring[2], Bernhard C. Lechtenberg[2,6], Taras V. Pogorelov[3], Kalina Hristova[1,4]*, Elena B. Pasquale[2]*

[1] Department of Materials Science and Engineering, Institute for NanoBioTechnology, Johns Hopkins University, Baltimore, MD 21218, USA

[2] Cancer Center, Sanford Burnham Prebys Medical Discovery Institute, La Jolla, CA 92037, USA

[3] Department of Chemistry, Center for Biophysics and Quantitative Biology, Beckman Institute for Advanced Science and Technology, and National Center for Supercomputing Applications, School of Chemical Sciences, University of Illinois at Urbana−Champaign, Urbana, Illinois 61801, USA

[4] Program in Molecular Biophysics, Institute for NanoBioTechnology, Johns Hopkins University, Baltimore, MD 21218, USA

[5] These authors contributed equally

[6] Current Address: Ubiquitin Signalling Division, The Walter and Eliza Hall Institute of Medical Research, Parkville Victoria 3052, Australia and Department of Medical Biology, The University of Melbourne, Parkville, Victoria 3010, Australia

* Correspondence: elenap@sbpdiscovery.org and kalina.hristova@jhu.edu

## SUPPORTING FIGURE LEGENDS

**Figure S1. Conformational fluctuations of the EphA4 and EphA2 SAM domains during MD simulations.** RMSD values were calculated for the backbone atoms of the EphA4 WT, EphA4 L920F, EphA2 WT and EphA2 L913F SAM domains, excluding the flexible termini. The crystal structures of the EphA4 SAM domain (residues 915-975, PDB ID: 1B0X) and of the EphA2 SAM domain (residues 909-969, PDB ID: 2KSO, chain A) were used as the reference structures. The time evolution of the RMSD values is plotted for each of the three MD simulation replicates (Rep 1-3) for each of the four SAM domains. The rapid, large variations in the RMSDs for the EphA4 L920F mutant suggest protein instability, while EphA4 WT, EphA2 WT, and the EphA2 L913F mutant generally exhibit smaller RMSD variations, with instabilities observed for only one of the repetitions for the EphA2 L913F mutant.

**Figure S2. Surface accessibility of residues in the simulated EphA4 WT and L920F SAM domains.** The solvent accessible surface area (SASA) was determined along the MD simulation trajectory for select residues W919, L920 or F920, F932, and H945 of the EphA4 WT and L920F SAM domains. The SASA trajectory for each of the four resides of the EphA4 WT SAM domain is compared to the corresponding residue of the EphA4 L920F SAM domain. The mean values and standard deviations are indicated in each panel,

**Figure S3. Deletion of the SAM domain has negligible effect on EphA4 WT dimerization and confirms the importance of the L920F mutant SAM domain.** (**A**) FRET efficiencies measured for the SAM domain-deleted (ΔSAM) EphA4 mutant in HEK293 cells compared to EphA4 WT. (**B**) The MSE values calculated for EphA4 ΔSAM. (**C**) Dimerization curve for EphA4 ΔSAM compared to EphA4 WT. (**D**) FRET efficiencies measured for EphA4 ΔSAM compared to EphA4 L920F. (**E**) Comparison of the oligomerization curves for EphA4 ΔSAM and EphA4 L920F demonstrates the key role of the L920F mutant SAM domain in promoting EphA4 oligomerization.

**Figure S4. Comparison of FSI-FRET and FIF data in the presence and absence of ligand.** FRET efficiencies as a function of acceptor concentration and dimerization/oligomerization curves as a function of total receptor concentration measured for (**A,B**) EphA4 WT, (**D,E**) EphA4 L920F and (**G,H**) EphA4 L920F-H945E in the presence and absence of ephrinA5-Fc. Comparison of FIF histograms for (**C**) EphA4 WT and (**F**) EphA4 L920F in the absence and presence of ephrinA5-Fc. The histogram maxima are indicated by the dotted lines. (**I**) High concentration FIF histograms comparing EphA4 WT (with ephrinA5-Fc ligand) and the L920F mutant (without ligand) demonstrate the different effects of the mutation and ligand binding.

**Figure S5. Histograms of RMSD values used to identify EphA4 SAM dimer decoy outliers.** The RMSD values from Fig. 6A,C were grouped into bins of size 0.3 and a gaussian curve was fit to each of the histograms for the EphA4 L920F dimer AB, EphA4 L920F dimer CD, and EphA4 WT SAM domain dimer decoys. The RMSD value at the Gaussian fit maximum is indicated in the top left corner of each plot along with the standard error. A SAM domain dimer decoy is considered an outlier if the RMSD value of that decoy deviates > 1 Å from the mean RMSD value. The models shown in Figs. 6B,D and S6 are within the selected 1 Å cutoff.

**Figure S6. EphA4 WT SAM domain dimer models.** (**A**) Structural representation of the lowest energy EphA4 WT dimer structure (Fig. 6C, green) where the two SAM domains (A and B) are indicated in green and gray, respectively. Residue L920 is indicated in blue and residue H945 in yellow, both shown as sticks and as a molecular surface. (**B**) Structural representation of the second lowest energy EphA4 WT dimer structure as shown in Fig. 6D. Residue L920 is indicated in blue and residue H945 in yellow, both shown as sticks/molecular surface. (**C**) Solved crystal structure of the EphA4 SAM domain (PDB ID: 1B0X) (1). Shown is a crystallographic dimer representing the most favorable SAM-SAM interaction. Coloring and residue representation as in B.

## REFERENCES


1. Stapleton, D., Balan, I., Pawson, T., and Sicheri, F. (1999) The crystal structure of an Eph receptor SAM domain reveals a mechanism for modular dimerization. *Nature structural biology* **6**, 44-49

**Table S1.** ***In silico*** **alanine scan mutagenesis of predicted EphA4 SAM domain interface residues**

| L920F dimer AB | | L920F dimer CD | |
|---|---|---|---|
| **residue** | **Δ I.S.** | **residue** | **Δ I.S.** |
| H945 | 3.91 | W919 | 4.04 |
| M946 | 3.57 | H945 | 3.99 |
| D918 | 3.39 | Q970 | 3.68 |
| Q978 | 3.21 | Q975 | 3.66 |
| Q975 | 3.20 | L966 | 3.51 |
| L966 | 3.12 | M946 | 3.02 |
| E941 | 2.90 | D918 | 2.54 |
| K924 | 2.85 | M972 | 2.46 |
| H961 | 2.74 | Q977 | 2.28 |
| Q977 | 2.30 | Q948 | 2.26 |

The 10 residues found to be most critical in each of the two interfaces are ordered according to the change in interface score (Δ I.S.) caused by mutating that residue to alanine. The starting interface scores (when the residues are not mutated to alanine) are -13.06 for L920F dimer AB and -13.04 for L920F dimer CD. Mutating H945 is predicted to be very destabilizing for both L920F interfaces, but not for the WT interface (not shown). Other residues predicted to destabilize both L920F interfaces, but not the WT interface, are highlighted in yellow.

Figure S1

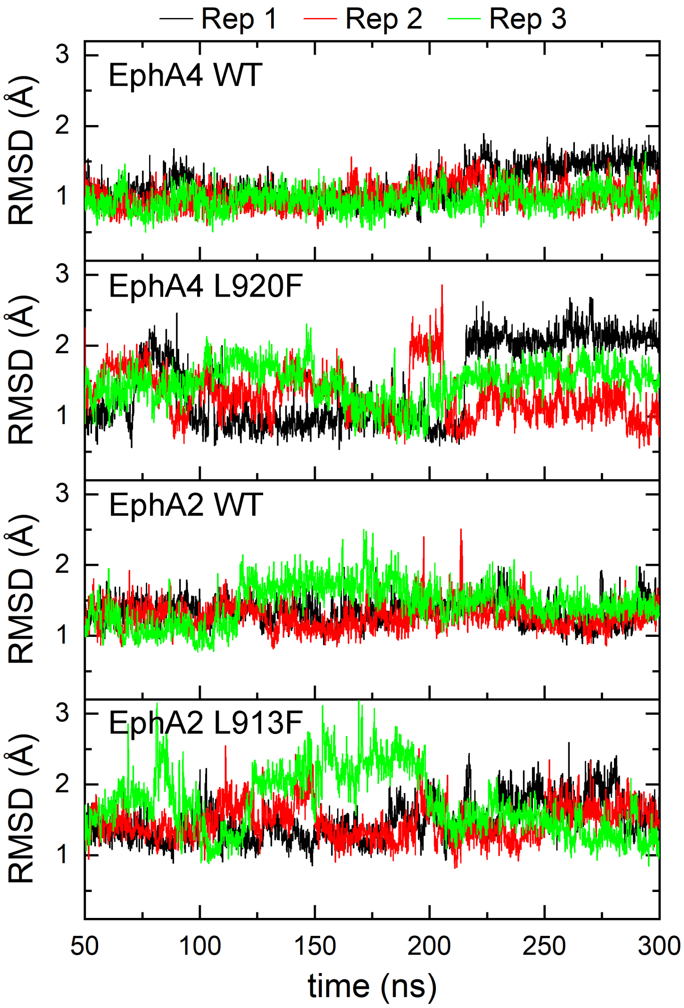

Rep 1
Rep 2
Rep 3
EphA4 WT
EphA4 L920F
EphA2 WT
EphA2 L913F
RMSD (Å)
1
2
3
50
100
150
200
250
300
time (ns)

Figure S2

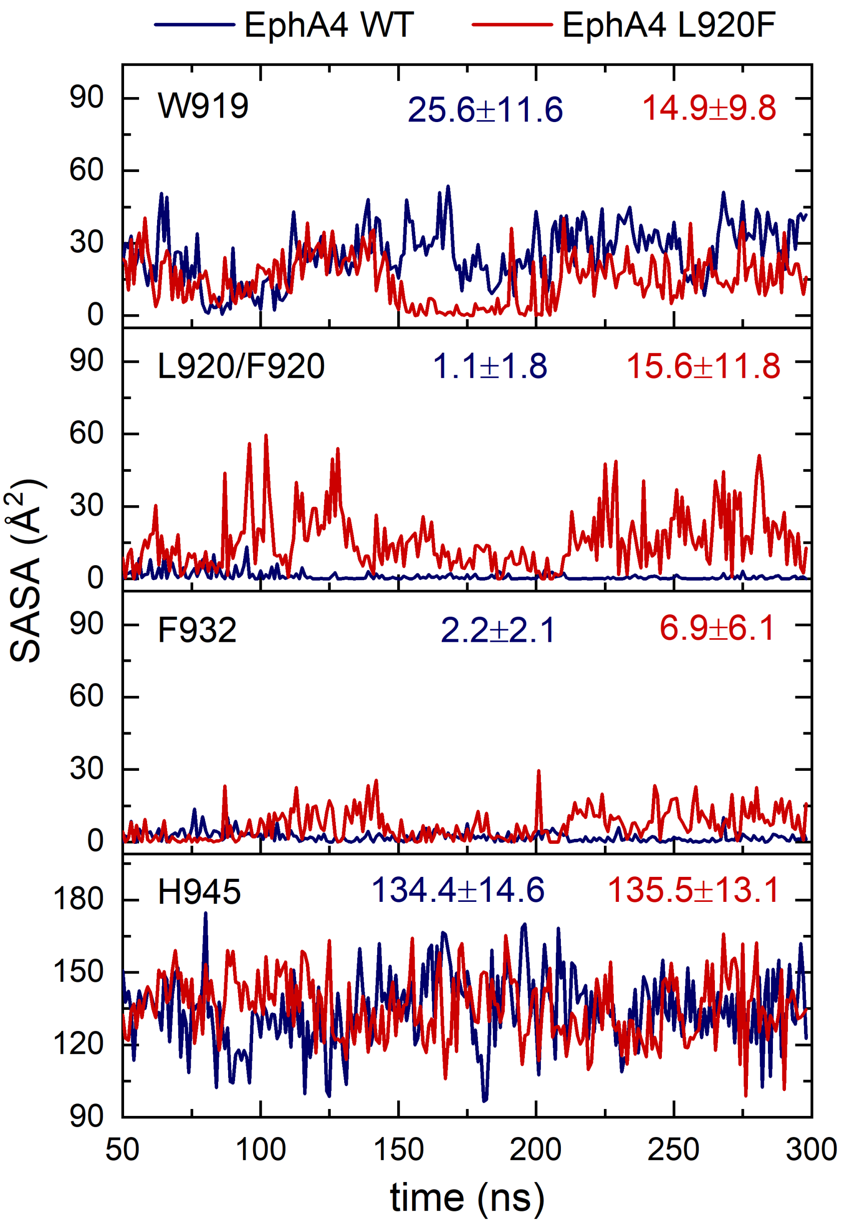

EphA4 WT
EphA4 L920F
W919
25.6±11.6
14.9±9.8
L920/F920
1.1±1.8
15.6±11.8
F932
2.2±2.1
6.9±6.1
H945
134.4±14.6
135.5±13.1
SASA (Å²)
time (ns)
0
30
60
90
120
150
180
50
100
150
200
250
300

Figure S3

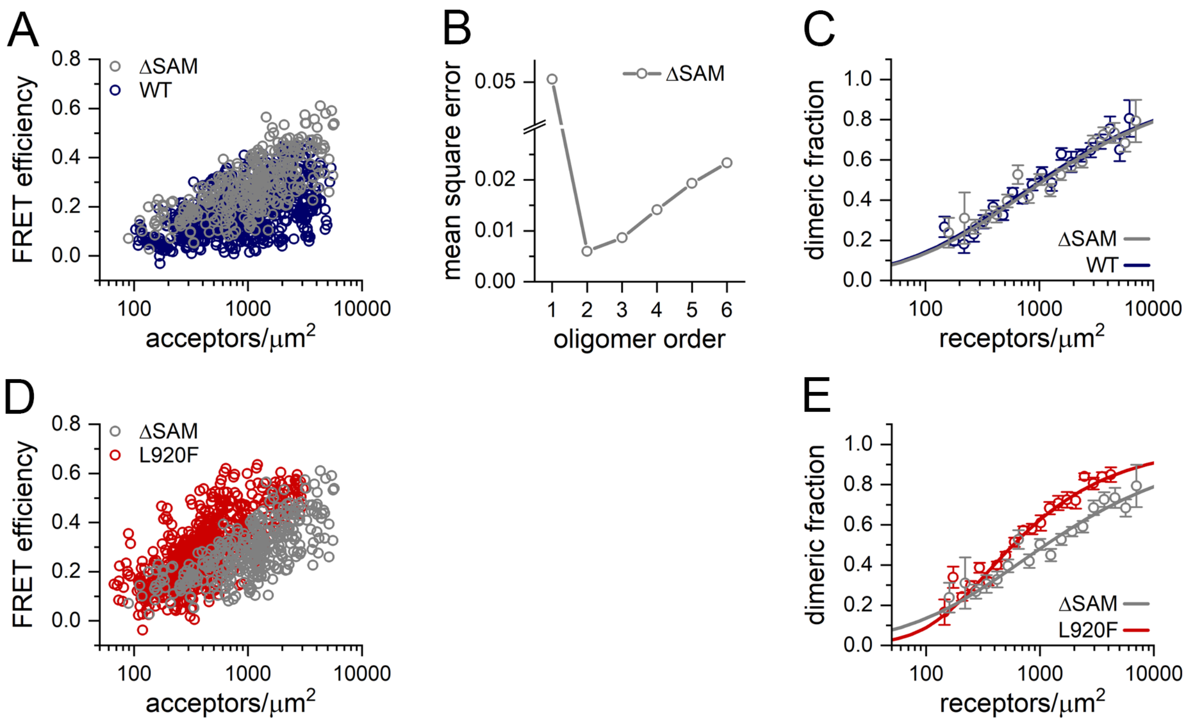

A
ΔSAM
WT
FRET efficiency
acceptors/μm²
B
ΔSAM
mean square error
oligomer order
C
dimeric fraction
receptors/μm²
ΔSAM
WT
D
ΔSAM
L920F
FRET efficiency
acceptors/μm²
E
dimeric fraction
receptors/μm²
ΔSAM
L920F

Figure S4

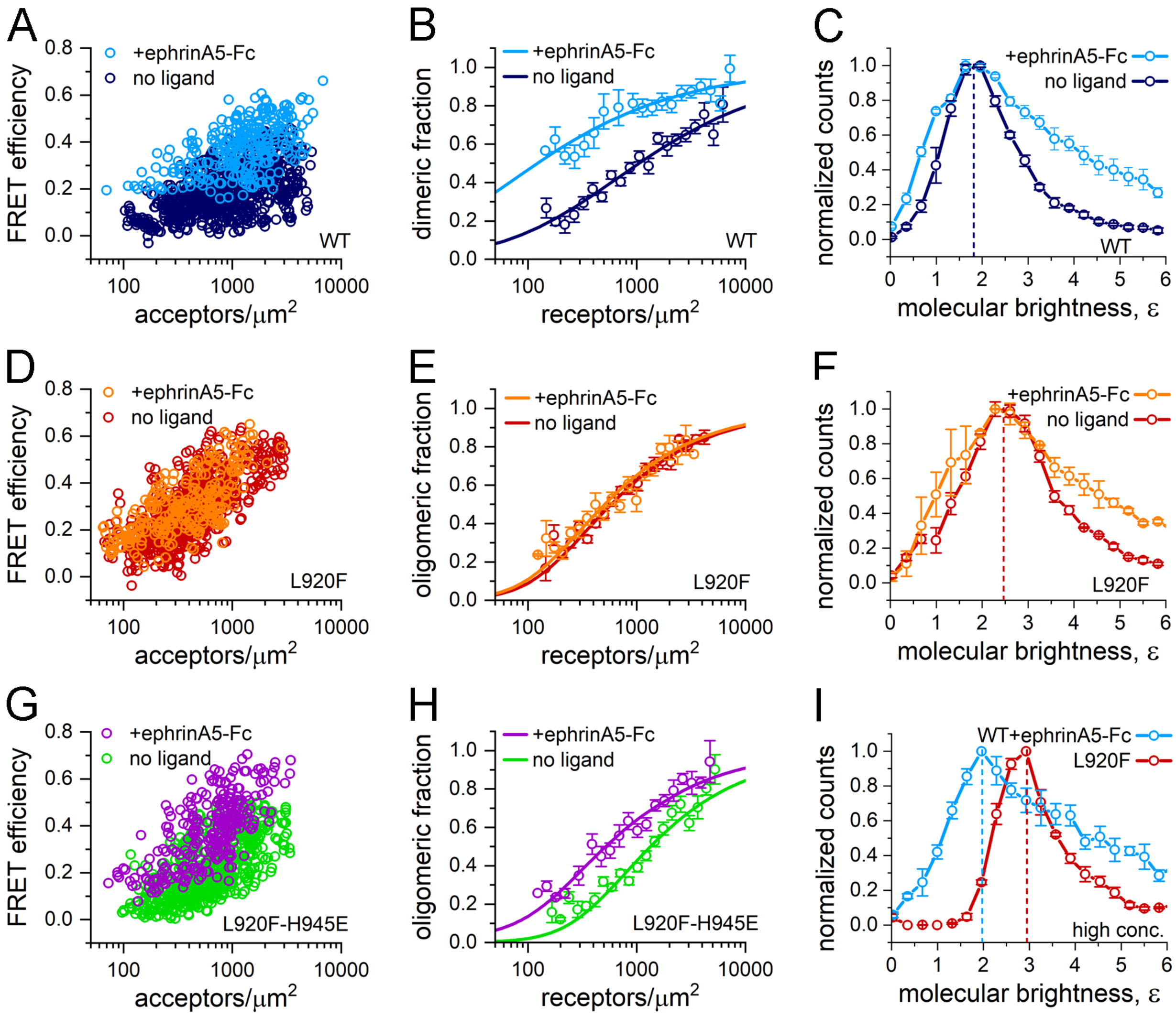
A
+ephrinA5-Fc
no ligand
FRET efficiency
WT
acceptors/μm2
B
+ephrinA5-Fc
no ligand
dimeric fraction
WT
receptors/μm2
C
+ephrinA5-Fc
no ligand
normalized counts
WT
molecular brightness, ε
D
+ephrinA5-Fc
no ligand
FRET efficiency
L920F
acceptors/μm2
E
+ephrinA5-Fc
no ligand
oligomeric fraction
L920F
receptors/μm2
F
+ephrinA5-Fc
no ligand
normalized counts
L920F
molecular brightness, ε
G
+ephrinA5-Fc
no ligand
FRET efficiency
L920F-H945E
acceptors/μm2
H
+ephrinA5-Fc
no ligand
oligomeric fraction
L920F-H945E
receptors/μm2
I
WT+ephrinA5-Fc
L920F
normalized counts
high conc.
molecular brightness, ε

Figure S5

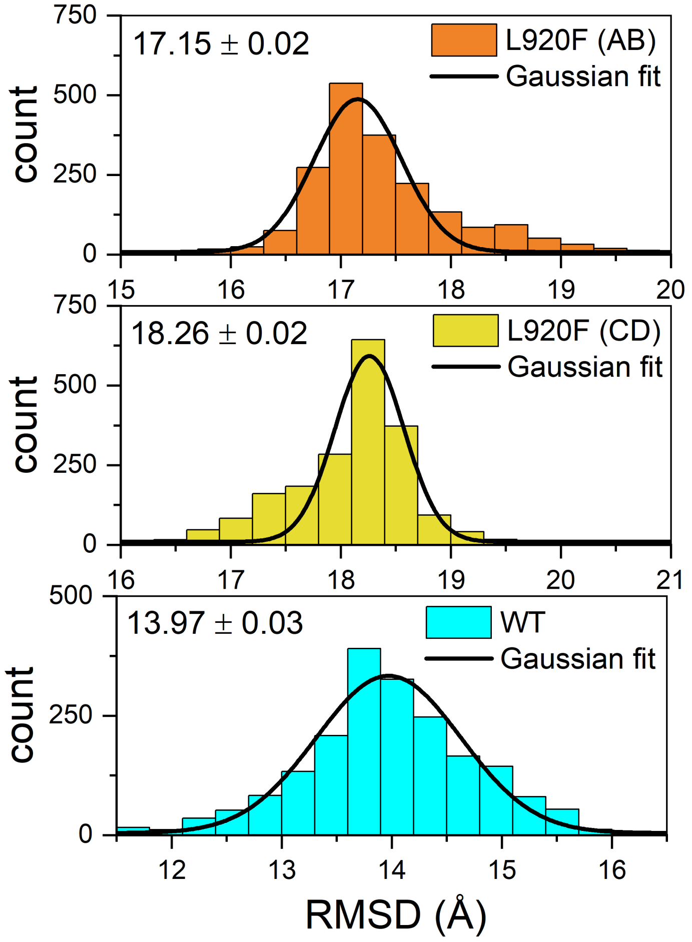

17.15 ± 0.02
L920F (AB)
Gaussian fit
count
750
500
250
0
15
16
17
18
19
20
18.26 ± 0.02
L920F (CD)
Gaussian fit
count
750
500
250
0
16
17
18
19
20
21
13.97 ± 0.03
WT
Gaussian fit
count
500
250
0
12
13
14
15
16
RMSD (Å)

Figure S6

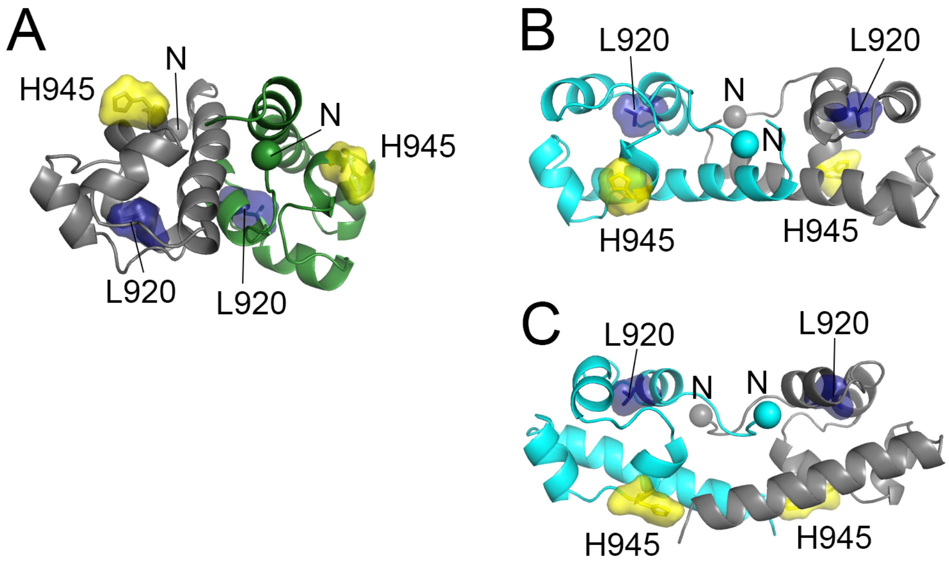

A
N
H945
N
H945
L920
L920
B
L920
L920
N
N
H945
H945
C
L920
L920
N
N
H945
H945
